# Mitogenomes of Nine Asian Skipper Genera and Their Phylogenetic Position (Lepidoptera: Hesperiidae: Pyrginae)

**DOI:** 10.3390/insects13010068

**Published:** 2022-01-06

**Authors:** Jintian Xiao, Jiaqi Liu, Luyao Ma, Xiangyu Hao, Ruitao Yu, Xiangqun Yuan

**Affiliations:** 1 Key Laboratory of Plant Protection Resources and Pest Management, Ministry of Education, Entomological Museum, College of Plant Protection, Northwest A&F University, Yangling, Xianyang 712100, China; xjt0629@nwafu.edu.cn (J.X.); jiaq_work@163.com (J.L.); mhjmly@163.com (L.M.); wrightyu@nwafu.edu.cn (R.Y.); 2 College of Life Sciences, Northwest A&F University, Yangling, Xianyang 712100, China; xyhao@nwafu.edu.cn

**Keywords:** mitochondrial genome, comparative genomics, phylogeny, Tagiadini, Celaenorrhini, *Capila*, *Pseudocoladenia*, *Sarangesa*

## Abstract

**Simple Summary:**

This is the first attempt to test the validity of morphological characters to diagnose the tribes used in the conventional taxonomy of the pyrginae skippers. Current studies on the group deal only with molecular phylogeny and its resulting classification without considering morphology. The diagnostic characters currently used in distinguishing the two tribes cannot be adopted. When a new taxonomic framework is proposed based on molecular data, reevaluation of morphological characters is suitable.

**Abstract:**

In this study, complete mitochondrial genomes of nine species representing three tribes in the subfamily Pyrginae *sensu lato* were newly sequenced. The mitogenomes are closed double-stranded circular molecules, with the length ranging from 15,232 bp to 15,559 bp, which all encode 13 protein-coding genes (PCGs), two ribosomal RNA (rRNA) genes, 22 transfer RNA (tRNA) genes, and a control region. The orientation and gene order of these nine mitogenomes are identical to the inferred ancestral arrangement of insects. All PCGs exhibit the typical start codon ATN except for *cox1* (using CGA) and *cox2* (using TTG) in *Mooreana trichoneura*. Most of the PCGs terminate with a TAA stop codon, while *cox1*, *cox2*, *nad4*, and *nad5* end with the incomplete codon single T. For the different datasets, we found that the one comprising all 37 genes of the mitogenome produced the highest nodal support, indicating that the inclusion of RNAs improves the phylogenetic signal. This study re-confirmed the status of *Capila*, *Pseudocoladenia,* and *Sarangesa*; namely, *Capila* belongs to the tribe Tagiadini, and *Pseudocoladenia* and *Sarangesa* to the tribe Celaenorrhini. Diagnostic characters distinguishing the two tribes, the length of the forewing cell and labial palpi, are no longer significant. Two populations of *Pseudocoladenia dan fabia* from China and Myanmar and *P. dan dhyana* from Thailand are confirmed as conspecific.

## 1. Introduction

The family Hesperiidae is one of species-richest groups of Lepidoptera, which account for one-fifth of the world’s butterfly species, though the number is significantly underestimated [1]. Traditionally, they had been classified into four or five subfamilies, and genus group was adopted instead of tribe, until the groups were divided [2] and members were re-classified based on molecular phylogeny [1,3]. Currently, as many as 10 subfamilies with numerous tribes are proposed [4,5].

The conventional concept of the subfamily Pyrginae is considered polyphyletic because members of the subfamily Eudaminae, which are morphologically significant, are often embedded within Pyrginae. As a result, Pyrginae is divided into three subfamilies: Tagiadinae (with the tribes Tagiadini, Netrocorynini, and Celaenorrhini), Pyrrhopygini (with the tribes Pyrrhopygini and Jerini), and Pyrginae (with the tribes Pyrgini, Carcharodini, Achlyodini, and Erynnini). These taxonomic arrangements, however, are purely or almost exclusively based on molecular data, and subfamily- and tribe-level assignments are arbitrary. On the one hand, biological taxonomy must reflect the phylogenetic relationship. On the other hand, however, it is a general reference system, and such a splitting of groups may result in taxonomic confusion. For this reason, herein we adopt conventional Pyrginae *sensu lato*, the subfamily currently composed of 646 species in 86 genera worldwide [6,7,8,9,10].

Evans in 1949 divided Pyrginae into the *Celaenorrhinus*-, *Tagiades*-, and *Pyrgus*-group, within the morphology-based taxonomic framework. He subsequently classified them into two sections [11]. The first section, characterized by erect labial palpi and long forewing cell, includes three genus groups: the *Augiades*-, *Urbanus*-, and *Celaenorrhinus*-group. The second section, characterized by porrect labial palpus and short forewing cell, also includes three genus groups: the *Telemiades*- (=*Tagiades*-), *Erynnis*-, and *Pyrgus*-group.

Most of the phylogenetic analyses of the family Hesperiidae rely on single-gene loci analyses, both mitochondrial (such as *cox1*, *cox2*, and *16S rRNA*) and nuclear (*wingless* and *EF-1α*). Although the mitogenome, in whole or part, has been widely used as a molecular marker in population genetics as well as evolutionary and phylogenetic studies, relatively few studies have attempted to study the phylogeny of Hesperiidae using the mitogenomes [12,13,14]. Data from a new perspective, such as complete mitogenomes, may help improve phylogenetic resolution for these groups. In this study, we sequenced nine additional mitogenomes of species belonging to nine genera in three tribes of the subfamily Pyrginae. In addition, we analyzed the characteristics of the nine mitogenomes in detail. Phylogenetic relationships of the family Hesperiidae were explored in combination with all the available 90 complete Pyrginae mitogenomic sequences available in GenBank. This enabled us to test the monophyly of Pyrginae, explore relationships within Pyrginae, and examine the taxonomic status of some ambiguous genera in Pyrginae.

Due to the compositional heterogeneity of the mitochondrial genome of Lepidopteran insects, most previous studies have attempted to address it by removing the third codon position of protein-coding genes, as well as excluding rRNAs and tRNAs [12,13,14,15,16,17]. The latest phylogenetic analysis by Ma et al. [18] established a dataset that contained RNAs and produced the most consistent topologies and higher node support values [19,20]. Therefore, the RNAs increased the phylogenetic resolution. Among the RNAs, tRNA is quite conservative, especially in the sequence of the stem area; its evolutionary rate is slower than the rate of other components of mitochondrial genomes. rRNAs also have higher conservatism and slower evolutionary rate. Thus, tRNA and rRNA are often used in phylogenetic analysis of various taxa, which has a great influence on the phylogenetic results [18,21,22,23,24]. Thus, we performed phylogenetic analysis of the family Hesperiidae using several different datasets to explore the impact of the inclusion or exclusion of tRNA on phylogenetic resolution.

Current studies deal only with molecular phylogeny and its resultant classification without reevaluating diagnostic morphological characters used in conventional taxonomy. We examined the wing venation and labial palpi of five genera to test the validity of those morphological characters as diagnostic features under the current phylogenetic framework.

## 2. Materials and Methods

### 2.1. Sample Collection and Genomic DNA Extraction

Mitochondrial genomes of nine species belonging to nine genera of Pyrginae were sequenced. Of those nine genera, three (*Satarupa*, *Mooreana*, and *Abraximorpha*) were newly sequenced genera and the rest were genera sequenced previously; therefore, we used different species in those genera, except for *Pseudocoladenia*, to confirm the monophyly of the genera. All the species/specimen used in this study are listed in (Table 1) and were collected and stored in 100% ethanol at −20 °C in the Entomological Museum, NWAFU. The specimens were initially examined using morphological characteristics, particularly the genitalia, and confirmed via *cox1* barcoding using the BOLD database [15,25,26]. The extraction of the Genomic DNA was done from the thoracic muscle (mitogenomes) using the Biospin Insect Genomic DNA Extraction Kit (Qiagen, Hilden, Germany). The NGS (Illumina HiSeq X; Biomarker Tech, Beijing, China) was employed to determine the nine mitogenomes of Pyrginae.

### 2.2. Bioinformatics Analyses

The extraction of the complete mitogenome sequences of the nine Pyrginae species was done using the Illumina HiSeq 2000 system by Genesky Biotechnologies Inc. (Shanghai, China). The correct identification rate of bases was very high, reaching 99.9%. First, the raw paired reads were retrieved and quality-trimmed using CLC Genomic Work bench v10.0 (CLC Bio, Aarhus, Denmark) with default parameters. The basic statistics of sequencing for each mitochondrial genome are presented in the Supplementary Materials (Table S1). The format of sequencing was Illumina, and the length of reads was 150 bp paired-ends. Then, the clean paired reads were used for mitogenome reconstruction using MITObim v1.7 software with default parameters and the mitogenome of *Tagiades vajuna* (KX865091) as the reference [16,27]. We selected a mitogenome, *T. vajuna*, as the reference and compared it with the nine mitogenomic sequences using MAFFT integrated into Geneious [28,29]. We conducted the annotation of the mitogenomes and comparative analyses following the methodology outlined above. We selected the complete mitogenome sequence of *T. vajuna* as a reference and used the Geneious 8.1.3 software to annotate all the various genomic features. Protein-coding genes (PCGs) were found by searching for ORFs (employing the invertebrate mitochondrial genetic code translation table5) and checking nucleotide alignments against the reference genome in Geneious 8.1.3. All RNAs (rRNAs and tRNAs) were identified using the MITOS Web Server (http://mitos.bioinf.uni-leipzig.de/index.py (accessed on 1 July 2021)), and tRNA secondary structures were visualized according to these results [27]. Finally, we used the Geneious software to visualize all gens by inspecting against the reference mitogenome. Nucleotide composition, codon usage, comparative mitogenomic architecture tables for the nine mitogenomes, and data used to plot RSCU (relative synonymous codon usage) figures were all calculated using PhyloSuite [28]. The nine newly sequenced mitogenome sequences were uploaded onto GenBank with the accession numbers as specified in Table 1.

### 2.3. Sequence Read Archive (SRA) Data Extraction

We downloaded and extracted the raw data of mitochondrial genomes of 86 hesperiid species from GenBank to reconstruct the phylogenetic relationships. The raw data were assembled and annotated by Geneious 8.1.3 [26]. The SRA data used in this study were obtained from GenBank and are listed in Table 2.

### 2.4. Phylogenetic Analysis

Phylogenetic analyses were conducted based on three datasets: (1) PCG: all codon positions of 13 protein-coding genes; (2) PRT: all codon positions of 13 protein-coding genes, 2 rRNAs, and 22 tRNAs; and (3) 12PRT: the first and second positions of 13 protein-coding genes, 2 rRNAs, and 22 tRNAs and two methods, (1) Bayesian inference (BI) and (2) maximum likelihood (ML), including 129 skipper species. The complete mitogenome genes were extracted using PhyloSuite v1.2.2, and the sequences of 13 PCGs of the 129 species were aligned in batches with MAFFT integrated into PhyloSuite. The best partitioning schemes and models for the Bayesian inference (BI) method and maximum likelihood (ML) method are specified in the Supplementary Materials (Tables S2 and S3). Nucleotide sequences were aligned using the G-INS-i (accurate) strategy and codon alignment mode. All rRNAs were aligned in the MAFFT with the Q-INS-i strategy [29]. Poorly matched sites in the alignments were removed using Gblocks v0.91b [30]. Individual genes were also concatenated using PhyloSuite v1.2.2.

### 2.5. Morphological Comparison

For the morphological comparison, we chose two types of the diagnostic characters one of which is the wing venation of five species representing five tribes. Another diagnostic characters is the lateral view of the head, showing the labial palpus of the five species representing five tribes. First, we rinsed the scales of Celanorrhinus maculosus and *Saranges dasahara* with 10% sodium hypochlorite and then traced the structure of the wing venation with digital boards, finally obtaining pictures with a simplified drawing. We examined the labial palpus of the five species using an electron microscope. Then, we constructed all the lateral views of head showing the labial palpus through freehand drawings.

## 3. Results and Discussion

### 3.1. Genome Organization and Base Composition

**Figure 1:**
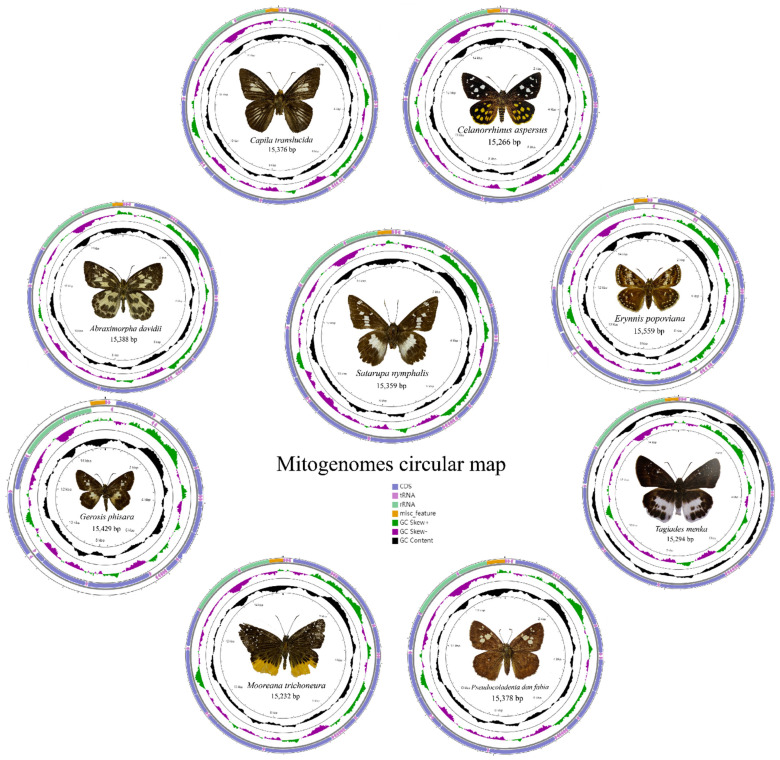
Circular maps of the mitogenomes of nine species.

The mitogenome of nine species are a single, covalently closed circular double-stranded DNA molecule (Figure 1) composed of 37 coding genes [48,49,50]. The mitogenome sizes are shown in Table 3. Including the newly sequenced mitogenomes of nine species in the present study, 49 species of Hesperiidae have mitogenome data public available, with the length ranging from 15,232 bp (*M. trichoneura*) to 15,559 bp (*Erynnis popoviana*).

All the nine mitogenomes contain 13 protein coding genes (PCGs), 22 transfer RNA genes (tRNAs), 2 ribosomal RNA genes (rRNAs), and an AT-rich region. Like many other insect mitogenomes, its major strand codes for 23 genes (9 PCGs and 14 tRNAs), while the minor strand codes for the remaining 14 genes (4 PCGs, 8 tRNAs, and 2 rRNA genes). The orientation and gene order of these mitogenomes of nine species are identical to the hypothesized ancestral arthropod arrangement found in the insect *Drosophila yakuba* [51].

The nucleotide composition of the nine mitogenomes is significantly biased towards A and T, with a relative A + T content of 79.5% to 82.4% in the whole genome, 77.5% to 80.9% in the PCGs, 81.2% to 83% in the transfer RNAs, and 84.3% to 86% in the ribosomal RNAs. Nearly all AT-skew and GC-skew are negative, except for the AT-skew in *Ce. aspersus* (0.003) and *Sat. nymphalis* (0.026), showing that there are more TC than AG across the whole mitogenome (Table 3).

### 3.2. Protein-Coding Genes

Of all the 13 PCGs in the nine mitogenomes of the subfamily Pyrginae, nine were located on the majority strand (J-strand), and the other four PCGs were located on the minority strand (N-strand). The total length of the 13 PCGs of nine species is 11,178 bp to 11,199 bp. The A + T content of the third codon positions of the PCGs (88–96.2%) was much higher than either the first (72.8–75.4%) or second codon positions (69.6–71.3%) (Table 4).

Most PCGs are initiated by a typical ATN as the start codon; the *cox1* of nine species have CGA as the start codon, and *cox2* in *M. trichoneura* is started with TTG. The canonical TAN stop codon occurs in most PCGs. In most of the nine species, *cox1*, *cox2*, *nad4*, and *nad5* genes use T as a truncated stop codon, except for the *nad4* gene of *P. dan fabia*, and *nad5* gene of *E. popoviana*, *M. trichoneura*, and *P. dan fabia*—all use TAA as a stop codon. Truncated stop codons are common in insect mitogenomes and might be completed by post-transcriptional polyadenylation [52].

**Figure 2:**
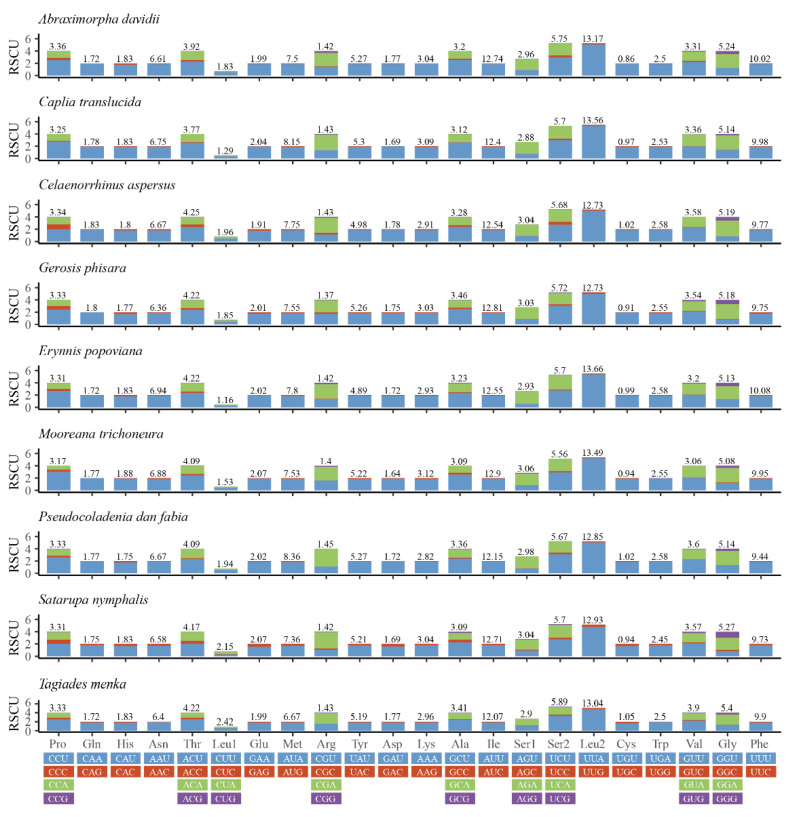
Relative synonymous codon usage (RSCU) in the mitogenomes of nine species.

Relative synonymous codon usage (RSCU) values for the nine mitogenomes are calculated and summarized in Figure 2. A + T bias is also reflected in the relative codon usage by the PCGs. The amino acid frequencies, excluding stop codons, are similar amongst the different skipper mitogenomes. The most frequently used codons across all the species are UUU (Phe), UUA (Leu), AUU (Ile), AUA (Met), and AAU (Asn), all of which are composed wholly of A or T. The results indicate a preference for NNU and NNA codons in skipper mitogenomes, which has been observed before [18,24,32].

### 3.3. Transfer and Ribosomal RNA Genes

All 22 standard tRNAs of the mitogenomes of the arthropod were found in the nine mitogenomes. The total length of tRNAs in the nine mitogenomes was 2121 bp (*A. davidii*) to 2165 bp (*Ce. aspersus*) (Table 3). Most tRNAs could be folded into the canonical cloverleaf structure, except for *trnS1* (AGN), with its dihydrouracil (DHU) arm forming a simple loop (all the secondary structure of tRNAs are shown in Supplementary Material Figures S1–S9), which was considered a typical feature in metazoan mitogenomes (Wolstenholme 1992). Fourteen tRNA genes were encoded by J-strand and the remaining eight were encoded by N-strand. There were base pair mismatches in the receptor arm, DHU arm, anticodon arm, and TΨC arm on the tRNAs of the nine mitogenomes, most of which were G-U mismatches, followed by U-U mismatches, and U-C, A-C, A-A, and A-G pairs (Table 5).

The two rRNA genes, *rrnL* and *rrnS*, were located between *trnL* (CUN) and *trnV*, and *trnV* and the A + T-rich region, respectively (Table 6). In the nine newly sequenced mitogenomes, the lengths ranged from 1347 bp (*A. davidii*) to 1390 bp (*M. trichoneura*) for the *rrnL*, and from 772 bp (*T. menaka*) to 788 bp (*M. trichoneura*) for the *rrnS* (Table 6). The A + T content of total rRNA genes was 84.3% (*Sat. nymphalis*) to 86% (*E. popoviana*), which was higher than that in the whole genome, indicating a moderate A + T preference in the total rRNA genes.

The control region (A + T-rich region) was located between *rrnS* and *trnM*, which ranged from 278 bp to 466 bp. This region contained the highest proportion of A and T, ranging from 92.7% to 96.3%. The high A+T could be involved in the regulation of transcription and replication of the mitogenome [52,53]. Both the AT-skew and GC-skew were negative in the control region of most species (except for the AT-skew of *Ce. aspersus*, *Sat. nymphalis*), indicating a clear bias towards the utilization of T and C.

### 3.4. Phylogenetic Relationships

**Figure 3:**
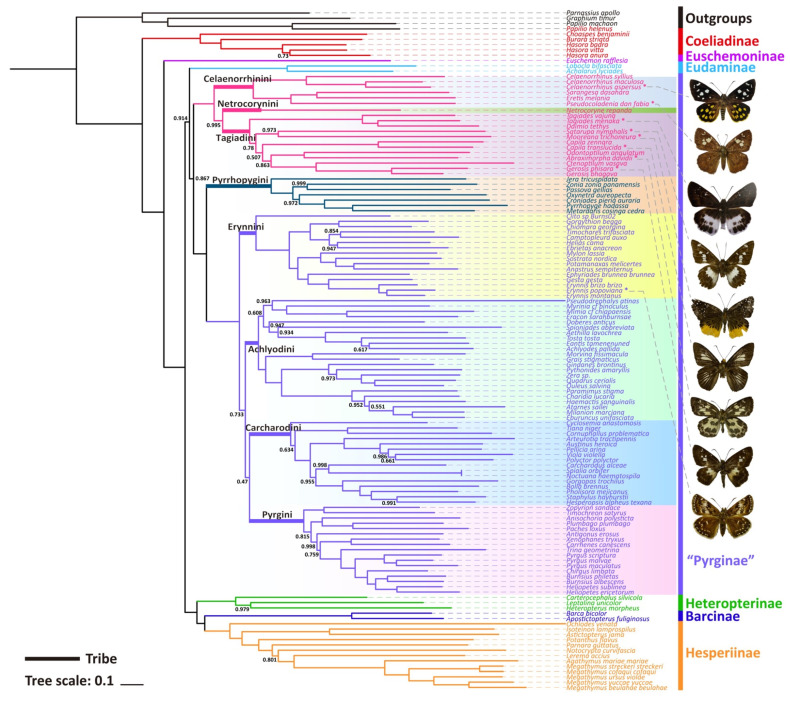
Phylogenetic tree produced by Mrbayes of PRT dataset. The bootstrap support (BS) values of 1 are hidden.

Only one phylogenetic hypothesis based on PRT datasets and using the BI method was proposed here (Figure 3); the rest are shown in the Supplementary Materials (Figures S10–S14). The resulting tree topologies are all congruent at the subfamily level, and nodal support values vary slightly for different analyses (Figure S15). The phylogenetic relationship among subfamilies is (Coeliadinae + (Euschemoninae + (Eudaminae + Pyrginae *sensu lato*) + (Heteropterinae + (Barcinae + Hesperiinae)))).

The monophyly of the subfamily Pyrginae is strongly supported (nodal support value is 0.867) as is the monophyly of Eudaminae + Pyrginae (nodal support value is 0.914), which means that these two subfamilies are not necessarily divided if based on this phylogeny. There are some differences in the phylogenetic topological structures obtained based on different datasets and different methods in this study, which are mainly reflected in the positional relationships of the subfamily Eudaminae, and the tribe Tagiadini and the phylogenetic relationships among the tribes Erynnini, Achlyodidini, Carcharodini, and Pyrgini. Since the BI tree based on PRT datasets has the high nodal support, only BI trees of PRT datasets are shown in this paper.

**Figure 4:**
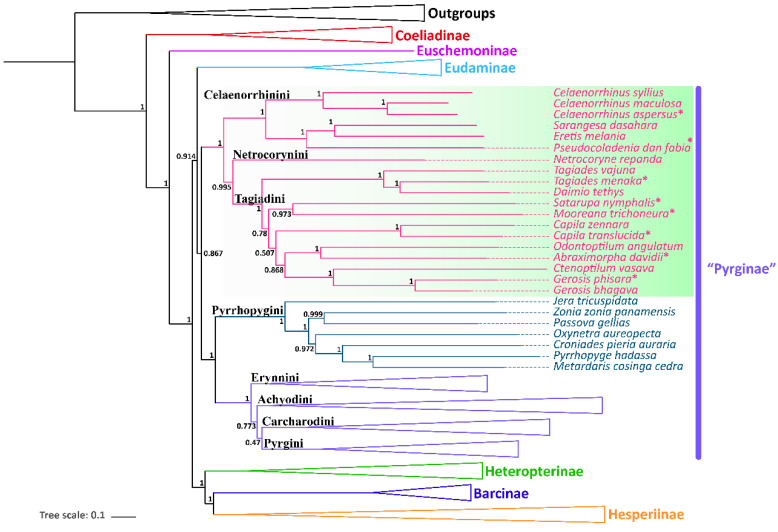
Phylogenetic tree produced by Mrbayes of PRT dataset. * indicates the materials added in this study.

All the nine samples are placed within the subfamily Pyrginae *sensu lato* (Figure 3 and Figure 4). As in the traditional taxonomy of the group, *Abraximorpha*, *Gerosis*, *Mooreana*, and *Tagiades* are placed in Tagiadini, *Celaenorrhinus* in Celaenorrhini, and *Erynnis* in Erynnini.

In the tribe Tagiadini, *Tagiades menaka* forms a monophyletic clade with *T. vajuna*. Our results support the view that the genus *Daimio Murray*, 1587, is a synonym of the genus *Tagiades* Hübner, 1819 [54]. The genus *Abraximorpha* is a sister group of the genus *Odontoptilum*. *Gerosis phisara* forms a monophyletic clade with *G. bhagava*. The genus *Ctenoptilum* and the genus *Gerosis* are sister groups with high nodal support and a stable relationship. *Abraximorpha*, *Odontoptilum*, and *Ctenoptilum* share a significant character, asymmetric genitalia. Based on our phylogeny, the character was lost secondarily in *Gerosis*. Another character also lost in *Gerosis* is a hair tuft at the tip of the female abdomen. The genus *Satarupa* forms a monophyletic clade with the genus *Mooreana*. In the tribe Celaenorrhini, *Celaenorrhinus aspersus* forms a monophyletic clade with *C. maculosus* and *C. syllius* with strong support. In the tribe Erynnini, *Erynnis popovina* forms a monophyletic clade with *E. montanus* and *E. brizo brizo*.

This study re-confirmed the status of *Capila*, *Pseudocoladenia*, and *Sarangesa*. *Capila* belongs to the tribe Tagiadini, and *Pseudocoladenia* and *Sarangesa* belong to the tribe Celaenorrhinini [5].

*Capila translucida* forms a monophyletic clade with *C. zennara* in the tribe Tagiadini. In Celaenorrhini, *Sarangesa* forms a monophyletic clade with *Eretis*. The intergeneric relationship within the tribe Celaenorrhinini is (*Celaenorrhinus* + ((*Sarangesa* + *Eretis*) + *Pseudocoladenia*)).

**Figure 5:**
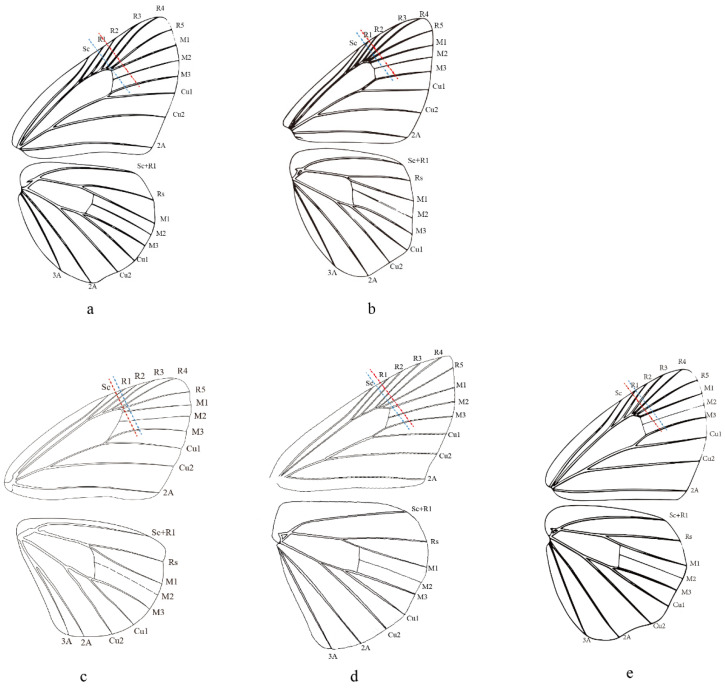
Wing venation of (**a**) *Tagiades vajuna*, (**b**) *Caplia omeia*, (**c**) *Celanorrhinus maculosus*, (**d**) *Saranges dasahara*, and (**e**) *Pseudocoladenia dan*. The blue dashed line denotes the length of the dorsun, and the red dashed line denotes 2/3 the length of the costa. (**a**,**b**,**e**) are reproduced from Chou (1998) [56].

Evans (1949) adopted the length of the forewing cell and labial palpi to distinguish his Celaenorrohinus-group and Tagiades-group. Then, Evans (1952) more precisely stated that the forewing cell is equal to two-thirds of the length of the costa and equal to or longer than the dorsum in Pyrginae Section I, including current Celaenorrhini. In contrast, the forewing cell of *Celaenorrhinus maculosus* (Figure 5c) is equal to the dorsum but much shorter than two-thirds of the costa. On the other hand, in *Tagiades vajuna* (Figure 5a), the forewing cell is shorter than two-thirds of the length of the costa but equal to the dorsum. In *Capila omeia* (Figure 5b), which is shifted from Celaenorrhini to Tagiadini, the forewing cell is not equal to or longer than two-thirds the length of the costa or dorsum. This is also the case in *Sarangesa dasahara* (Figure 5d) and *Pseudocoladenia dan* (Figure 5e), both of which are shifted from Tagiadini to Celaenorrhinni. To summarize, the length of the forewing cell cannot be adopted as a diagnostic character to distinguish the tribes Celaenorrhini and Tagiadini.

**Figure 6:**
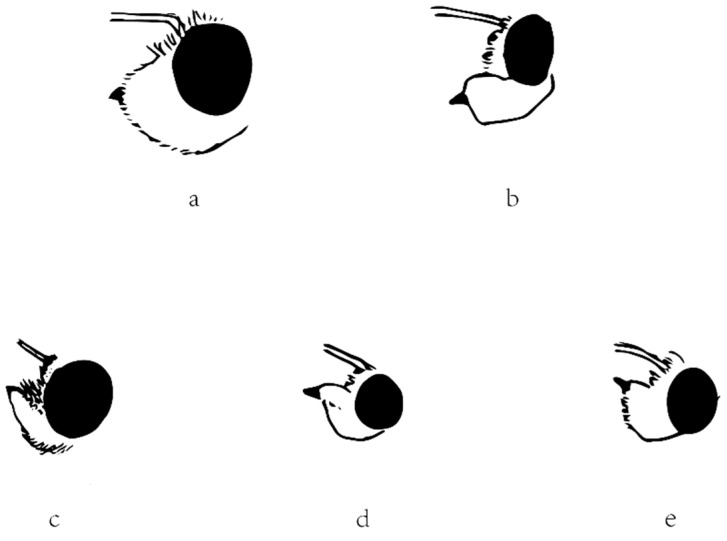
Lateral view of head showing labial palpus of (**a**) *Tagiades menaka*, (**b**) *Capila jayadeva*, (**c**) *Celaenorrhinus maculosus*, (**d**) *Sarangesa dasahara*, and (**e**) *Pseudocoladenia dan*. The ratio between Figure 6 and real specimens is 2.5:1.

The other diagnostic character that Evans (1949, 1952) used is the labial palpi. In Section I in America or the Celaenorrhinus-group in Asia, the labial palpi are erect. In other words, the second segment is appressed to the face, and the third segment is not protruding in front of the second segment. However, in Section II in America or the Tagiades-group in Asia, the labial palpi aren’t erect, and the third segment is protruding. The character is obviously applicable for *Celaenorrhinus* (Figure 6c) and *Tagiades* (Figure 6a), respectively. In *Capila* (Figure 6b), shifted from Celaenorrhini to Tagiadini, the third segment protrudes in front of the second segment, but this is also the case in *Sarangesa* (Figure 6d) and *Pseudocolodenia* (Figure 6e), shifted the other way. Therefore, in conclusion, the character cannot be adopted for either. Thus, we were not successful in finding a good diagnostic character for distinguishing between these two tribes.

Evans (1949) classified *Coladenia* (now *Pseudocoladenia*) *dan* into 11 subspecies in four groups. Subsequently, because of their sympatric distribution and the differences in male and female genitalia, Huang and Xue (2004) raised the taxonomic status of two taxa from China to distinct species. In order to clarify the species limitation of some taxa, we calculated p-distance between our sample, *P. dan fabia* from Yunnan Province, China, and that from Myanmar (GenBank #SRR7174480) and *P. dan dhyana* from southern Thailand (#KY019868). The distance between *P. dan fabia* from the two different localities was 0.022, whereas the distance between *P. dan fabia* from China and *P. dan dhyana* was 0.027. Though the latter number reaches around the border of species and subspecies [55], we retained the taxonomic status, pending inclusion of the nominate subspecies dan from South India in our analysis.

## Figures and Tables

**Table 1:** Species information and GenBank accession numbers.

Species	Accession Number	Collection Spot
*Abraximorpha davidii*	MZ221157	Zhoushan, Zhejiang, China
*Capila translucida*	MZ221158	Jianfengling, Hainan, China
*Celaenorrhinus aspersus*	MZ221159	Guanyang County, Guangxi, China
*Erynnis popoviana*	MZ221165	Huangling County, Shaanxi, China
*Gerosis phisara*	MZ221160	Mountain Fengyangshan, Zhejiang, China
*Mooreana trichoneura*	MZ221161	Yexianggu, Yunnan, China
*Pseudocoladenia dan fabia*	MZ221162	Mengla County, Yunnan, China
*Satarupa nymphalis*	MZ221163	Xianxialing, Zhejiang, China
*Tagiades menaka*	MZ221164	Jianfengling, Hainan, China

**Table 2:** Species information and GenBank accession numbers.

Subfamily	Tribes	Species	Accession Number	References
Coeliadinae		*Burara striata*	NC_034676	[31]
		*Choaspes benjaminii*	NC_024647	[32]
		*Hasora anura*	KF881049	[33]
		*Hasora vitta*	NC_027170	[34]
		*Hasora badra*	NC_045249	Unpublished
Euschemoninae		*Euschemon rafflesia*	NC_034231	[35]
Heteropterinae		*Carterocephalus silvicola*	NC_024646	[32]
		*Heteropterus morpheus*	NC_028506	Unpublished
		*Leptalina unicolour*	MK265705	[36]
Barcinae		*Apostictopterus fuliginosus*	NC_0339946	[24]
		*Barca bicolor*	NC_0339947	[24]
Hesperiinae		*Lerema accius*	NC_029826	[37]
		*Ochlodes venata*	HM243593	Unpublished
		*Parnara guttata*	NC_029136	[38]
		*Potanthus flavus*	KJ629167	[32]
		*Astictopterus jama*	MH763663	[18]
		*Isoteinon lamprospilus*	MH763664	[18]
		*Notocrypta curvifascia*	MH763665	[18]
		*Agathymus mariae mariae*	KY630504	[39]
		*Megathymus beulahae beulahae*	KY630505	[39]
		*Megathymus cofaqui cofaqui*	KY630503	[39]
		*Megathymus streckeri streckeri*	KY630501	[39]
		*Megathymus ursus violae*	KY630502	[39]
		*Megathymus yuccae yuccae*	KY630500	[39]
Eudaminae		*Achalarus lyciades*	NC_030602	[40]
		*Lobocla bifasciata*	KJ629166	[32]
Pyrginae	Celaenorrhini	*Celaenorrhinus maculosus*	NC_022853	[41]
		*Celaenorrhinus syllius*	SRR7174479	[5]
		*Celaenorrhinus aspersus*	MZ221157	This study
		*Eretis melania*	SRR7174485	[5]
		*Pseudocoladenia dan fabia*	MZ221162	This study
		*Sarangesa dasahara*	SRR7174486	[5]
	Netrocorynini	*Netrocoryne repanda*	SRR7174483	[5]
	Tagiadini	*Abraximorpha davidii*	MZ221157	This study
		*Capila translucida*	MZ221157	This study
		*Capila zennara*	SRR7174484	[5]
		*Ctenoptilum vasava*	JF713818	[42]
		*Tagiades (=Daimio) tethys*	KJ813807	[43]
		*Gerosis bhagava*	SRR7174473	[5]
		*Gerosis phisara*	MZ221157	This study
		*Mooreana trichoneura*	MZ221162	This study
		*Satarupa nymphalis*	MZ221162	This study
		*Tagiades menaka*	MZ221157	This study
		*Tagiades vajuna*	KX865091	[16]
	Pyrrhopygini	*Croniades pieria auraria*	SRR7174434	[5]
		*Jera tricuspidata*	SRR7174433	[5]
		*Metardaris cosinga cedra*	SRR7174435	[5]
		*Odontoptilum angulatum*	MW381783	[44]
		*Oxynetra aureopecta*	SRR7174437	[5]
		*Passova gellias*	SRR7174439	[5]
		*Pyrrhopyge hadassa*	SRR7174436	[5]
		*Zonia zonia panamensis*	SRR7174438	[5]
	Erynnini	*Anastrus sempiternus*	SRR7174507	[5]
		*Camptopleura auxo*	SRR7174462	[5]
		*Clito* sp.	SRR7174503	[5]
		*Chiomara georgina*	SRR7174467	[5]
		*Ebrietas anacreon*	SRR7174464	[5]
		*Ephyriades brunnea brunnea*	SRR7174465	[5]
		*Erynnis brizo brizo*	SRR7174469	[5]
		*Erynnis montanus*	NC_021427	[32]
		*Erynnis popoviana*	MZ221162	This study
		*Gesta gesta*	SRR7174466	[5]
		*Gorgythion begga*	SRR7174468	[5]
		*Helias cama*	SRR7174506	[5]
		*Mylon lassia*	SRR7174502	[5]
		*Potamanaxas melicertes*	SRR7174504	[5]
		*Sostrata nordica*	SRR7174505	[5]
		*Timochares trifasciata*	SRR7174461	[5]
	Achyodini	*Aethilla lavochrea*	SRR7174432	[5]
		*Achlyodes pallida*	SRR7174366	[5]
		*Atarnes sallei*	SRR7174338	[5]
		*Charidia lucaria*	SRR7174567	[5]
		*Doberes anticus*	SRR7174369	[5]
		*Eantis tamenund*	SRR7174365	[5]
		*Eburuncus unifasciata*	SRR7174345	[5]
		*Eracon sarahburnsae*	SRR7174373	[5]
		*Gindanes brontinus*	SRR7174337	[5]
		*Grais stigmaticus*	SRR7174368	[5]
		*Haemactis sanguinalis*	SRR7174339	[5]
		*Milanion marciana*	SRR7174344	[5]
		*Mimia* cf. *chiapaensis*	SRR7174372	[5]
		*Morvina fissimacula*	SRR7174367	[5]
		*Myrinia* cf. *binoculus*	SRR7174371	[5]
		*Ouleus salvina*	SRR7174340	[5]
		*Paramimus stigma*	SRR7174568	[5]
		*Pseudodrephalys atinas*	SRR7174341	[5]
		*Pythonides amaryllis*	SRR7174336	[5]
		*Quadrus cerialis*	SRR7174343	[5]
		*Spioniades abbreviata*	SRR7174370	[5]
		*Tosta tosta*	SRR7174431	[5]
		*Zera* sp.	SRR7174342	[5]
	Carcharodini	*Arteurotia tractipennis tractipennis*	SRR7174564	[5]
		*Austinus heroica*	SRR7174561	[5]
		*Bolla brennus*	SRR7174539	[5]
		*Carcharodus alceae*	SRR7174533	[5]
		*Cornuphallus problematica*	SRR7174563	[5]
		*Cyclosemia anastomosis*	SRR7174565	[5]
		*Gorgopas trochilus*	SRR7174532	[5]
		*Hesperopsis alpheus texana*	SRR7174536	[5]
		*Noctuana haematospila*	SRR7174535	[5]
		*Pellicia arina*	SRR7174570	[5]
		*Pholisora mejicanus*	SRR7174538	[5]
		*Polyctor polyctor*	SRR7174562	[5]
		*Spialia orbifer*	SRR7174534	[5]
		*Staphylus hayhurstii*	SRR7174537	[5]
		*Tiana niger*	SRR7174566	[5]
		*Viola violella*	SRR7174571	[5]
	Pyrgini	*Anisochoria polysticta*	SRR7174531	[5]
		*Antigonus erosus*	SRR7174496	[5]
		*Burnsius albescens*	SRR7174499	[5]
		*Burnsius phIletas*	SRR7174498	[5]
		*Carrhenes canescens*	SRR7174490	[5]
		*Chirgus limbata*	SRR7174488	[5]
		*Heliopetes ericetorum*	SRR7174500	[5]
		*Heliopetes sublinea*	SRR7174501	[5]
		*Plumbago plumbago*	SRR7174495	[5]
		*Paches loxus*	SRR7174494	[5]
		*Pyrgus malvae*	SRR7174492	[5]
		*Pyrgus scriptura*	SRR7174487	[5]
		*Pyrgus maculatus*	NC_030192	Unpublished
		*Timochreon satyrus*	SRR7174493	[5]
		*Trina geometrina*	SRR7174491	[5]
		*Xenophanes tryxus*	SRR7174489	[5]
		*Zopyrion sandace*	SRR7174530	[5]
Outgroup				
Papilionidae		*Papilio machaon*	NC_018047	Unpublished
		*Papilio helenus*	NC_025757	[45]
		*Graphium timur*	NC_024098	[46]
		*Parnassius apollo*	NC_024727	[47]

**Table 3:** Nucleotide composition and skewness of the nine mitogenomes.

Species	Whole Genome				PCGs				rRNA				tRNA				A + T-Rich Region			
	Size (bp)	AT%	AT Skew	GC Skew	Size (bp)	AT%	AT Skew	GC Skew	Size (bp)	AT%	AT Skew	GC Skew	Size (bp)	AT%	AT Skew	GC Skew	Size (bp)	AT%	AT Skew	GC Skew
*A. davidii*	15,388	81.4	−0.018	−0.211	1480	82.5	0.025	0.181	11,190	79.7	−0.16	0.009	2121	85.2	0.028	0.327	278	96.1	−0.086	−0.273
*Ca. translucida*	15,376	82.4	−0.027	−0.186	1467	83	0.015	0.165	11,181	80.9	−0.15	0.038	2143	85.5	0.027	0.327	333	96.3	−0.022	−0.333
*Ce. aspersus*	15,266	80.4	0.003	−0.22	1458	81.7	0.013	0.184	11,178	78.9	−0.146	0	2165	84.6	−0.016	0.373	333	93.6	0.019	−0.333
*E. popoviana*	15,559	81.8	−0.003	−0.168	1465	82.1	0.013	0.172	11,190	80.3	−0.146	0.032	2156	86	0.009	0.307	368	93	−0.023	−0.231
*G. phisara*	15,429	80.4	−0.02	−0.212	1467	81.5	−0.001	0.14	11,199	78.6	−0.151	0.003	2144	85.2	0.058	0.308	391	94.9	−0.024	−0.2
*M. trichoneura*	15,232	81.7	−0.023	−0.191	1465	83	0.02	0.157	11,190	80.2	−0.152	0.011	2162	85.2	0.015	0.312	355	94.6	−0.06	−0.368
*P. dan fabia*	15,378	81.4	−0.024	−0.186	1460	82.2	0.01	0.131	11,190	79.9	−0.149	0.03	2151	84.7	0.018	0.331	466	94.7	−0.02	−0.2
*Sat. nymphalis*	15,359	79.5	0.026	−0.227	1481	81.2	0.024	0.151	11,190	77.5	−0.149	0.01	2143	84.3	−0.021	0.335	332	95.2	0	−0.375
*T. menaka*	15,294	80.2	−0.012	−0.215	1448	81.6	0.015	0.173	11,184	78.4	−0.175	0.007	2147	84.5	0.028	0.345	355	94.7	0.024	−0.263

**Table 4:** Base content of protein-coding gene.

Species	Regions	Size (bp)	T (U)	C	A	G	AT (%)
*A. davidii*	PCGs	11,190	46.2	10	33.5	10.2	79.7
	1st	3730	37.7	10.1	36.7	15.5	74.4
	2nd	3730	48.4	16.2	22.4	12.9	70.8
	3rd	3730	52.5	3.8	41.4	2.2	93.9
*Ca. translucida*	PCGs	11,181	46.5	9.2	34.4	9.9	80.9
	1st	3727	38.2	9.6	36.9	15.3	75.1
	2nd	3727	48.6	15.8	22.7	12.9	71.3
	3rd	3727	52.7	2.3	43.5	1.6	96.2
*Ce. aspersus*	PCGs	11,178	45.2	10.6	33.7	10.6	78.9
	1st	3726	36.9	10.3	37.1	15.7	74
	2nd	3726	48.2	16.5	22.1	13.2	70.3
	3rd	3726	50.5	4.9	41.8	2.8	92.3
*E. popoviana*	PCGs	11,190	46	9.5	34.3	10.2	80.3
	1st	3730	38.1	9.4	37.3	15.3	75.4
	2nd	3730	48.3	16.4	22.3	13	70.6
	3rd	3730	51.6	2.8	43.3	2.2	94.9
*G. phisara*	PCGs	11,199	45.2	10.7	33.4	10.8	78.6
	1st	3733	37.1	10.1	36.9	15.9	74
	2nd	3733	48.1	16.7	22.2	13	70.3
	3rd	3733	50.4	5.3	41	3.3	91.4
*M. trichoneura*	PCGs	11,190	46.2	9.8	34	10	80.2
	1st	3730	37.9	9.7	37.5	14.9	75.4
	2nd	3730	48.3	15.9	22.8	13	71.1
	3rd	3730	52.4	3.8	41.8	2.1	94.2
*P. dan fabia*	PCGs	11,190	45.9	9.8	34	10.4	79.9
	1st	3730	37	10.2	37	15.8	74
	2nd	3730	48.2	16.4	22.3	13.1	70.5
	3rd	3730	52.4	2.7	42.8	2.1	95.2
*Sat. nymphalis*	PCGs	11,190	44.5	11.1	33	11.3	77.5
	1st	3730	37.1	10.4	36.8	15.7	73.9
	2nd	3730	48.3	16.2	22.4	13.1	70.7
	3rd	3730	48.2	6.7	39.8	5.3	88
*T. menaka*	PCGs	11,184	46.1	10.7	32.3	10.9	78.4
	1st	3728	37.7	10.7	35.1	16.4	72.8
	2nd	3728	47.9	16.8	22	13.3	69.9
	3rd	3728	52.6	4.6	39.8	3	92.4

**Table 5:** Mismatch among the tRNAs of the nine mitogenomes.

Species	Acceptor Arm			DHU Arm		Anticodon Arm						TΨC Arm		
	G-U	U-U	U-C	G-U	U-U	G-U	U-U	A-C	U-C	A-A	A-G	G-U	A-C	U-U
*A. davidii*	5	2	1	3		2	1	2			1	2		
*Sat. nymphalis*	3	3		6		3	2	2				1		
*M. trichoneura*	3	4		6		2	2	2		1		2	1	
*P. dan fabia*	5	3		3		2	2	2						
*G. phisara*	4	2		7		3	3	2						1
*T. menaka*	5	2		8		5	2	1	1			2		
*E. popoviana*	4	3		6	1	3	2	1	1			3		
*Ca. translucida*	3	3		6	1	3	2	1	1			1		
*Ce. aspersus*	6	3		7	1	1	3	2				3		

**Table 6:** Mitogenomic organization of the nine mitogenomes.

Gene	Size	Intergenic Nucleotides	Codon		Strand
			Start	Stop	
*T. menaka*/*A. davidii*/*Ca. translucida*/*Ce. aspersus*/*E. popoviana*/*G. phisara*/*M. trichoneura*/*P. dan fabia*/*Sat. nymphalis*					
*trnM*	68/68/67/68/68/68/69/68/67				+
*trnI*	64/70/67/64/65/64/64/65/67	7/-/-/-/6/8/-/-1/10			+
*trnQ*	69/69/69/69/69/69/69/69/69	-3/-3/-3/1/-3/-3/8/-3/9/-3			-
*nad2*	1014/1014/1014/1014/1014/1014/1014/1014/1014	59/90/97/62/60/66/65/61/62/101	ATT/ATT/ATT/ATT/ATT/ATT/ATC/ATT/ATC	TAA/TAA/TAA/TAA/TAA/TAA/TAA/TAA/TAA	+
*trnW*	67/67/68/67/67/67/68/67	-2/-2/-2/-2/-2/-2/1/-1/-2/-2			+
*trnC*	67/65/63/66/67/69/66/69/68	-8/-8/-8/-8/-8/-8/-8/-8/-8/-8			-
*trnY*	65/66/64/66/65/67/66/65/75	-/3/8/11/1/1/26/2/3/8			-
*cox1*	1531/1531/1531/1531/1531/1531/1531/1531/1531	5/3/9/2/5/6/11/8/18/8	CGA/CGA/CGA/CGA/CGA/CGA/CGA/CGA/CGA	T/T/T/T/T/T/T/T/T	+
*trnL2*	67/67/67/67/67/67/70/67/67				+
*cox2*	676/673/676/676/679/676/673/676/676	-/-/-/-/-/-/1/-/-/-	ATT/ATG/ATG/ATG/ATG/ATG/TTG/ATG/ATG	T/T/T/T/T/T/T/T/T	+
*trnK*	71/71/71/71/71/71/70/71/71				+
*trnD*	67/73/71/67/69/71/66/67/67	7/7/19/22/4/2/7/-/26/17			+
*atp8*	168/165/159/162/162/171/159/162/159		ATC/ATT/ATT/ATT/ATT/ATC/ATC/ATT/ATT	TAA/TAA/TAA/TAA/TAA/TAA/TAA/TAG/TAA	+
*atp6*	678/678/678/678/678/678/678/678/678	-7/-7/-7/-7/-7/-7/-7/-7/-7/-7	ATG/ATG/ATG/ATG/ATG/ATG/ATG/ATG/ATG	TAA/TAA/TAA/TAA/TAA/TAA/TAA/TAA/TAA	+
*cox3*	786/786/786/786/786/777/786/786/786	-1/-1/-1/-1/-1/-1/-1/-1/-1/-1	ATG/ATG/ATG/ATG/ATG/ATG/ATG/ATG/ATG	TAA/TAA/TAA/TAA/TAA/TAA/TAA/TAA/TAA	+
*trnG*	66/67/68/66/65/66/66/65/67	2/2/2/2/2/2/2/2/2/2			+
*nad3*	354/354/354/354/354/354/354/354/354		ATT/ATT/ATT/ATT/ATT/ATT/ATT/ATT/ATT	TAA/TAA/TAA/TAA/TAA/TAA/TAA/TAA/TAA	+
*trnA*	64/64/65/66/68/69/65/67/67	19/-/17/9/4/16/11/5/6/3			+
*trnR*	64/67/62/63/63/64/66/67/66	7/1/-/-1/1/3/-1/-1/-1/19			+
*trnN*	66/67/66/66/63/66/65/66/67	-/6/-/30/-/29/-1/-3/-/-1			+
*trnS1*	61/62/62/60/67/61/62/60/62	14/9/112/3/3/21/5/4/-/2			+
*trnE*	66/70/69/68/68/66/66/66/67	-/9/5/1/9/9/15/2/-/8			+
*trnF*	67/68/67/63/66/66/67/65/67	18/-2/4/22/-2/-2/24/-2/-/-2			-
*nad5*	1747/1750/1750/1738/1743/1756/1755/1743/1741	2/-/-/-/-/121/-/3/1/-	ATT/ATT/ATT/ATT/ATT/ATA/ATT/ATT/ATA	T/T/T/T/TAA/T/TAA/TAA/T	-
*trnH*	65/70/72/65/68/67/66/66/68				-
*nad4*	1339/1339/1339/1339/1339/1339/1336/1341/1339	-/-/-/-/-/-/-/-/-1/-	ATG/ATG/ATG/ATG/ATG/ATG/ATG/ATG/ATG	T/T/T/T/T/T/T/TAA/T	-
*nad4L*	282/282/282/285/285/282/285/285/282	2/-/-1/6/2/2/-1/-7/4/-1	ATG/ATG/ATG/ATG/ATG/ATG/ATG/ATG/ATG	TAA/TAA/TAA/TAA/TAA/TAA/TAA/TAA/TAA	-
*trnT*	64/65/64/65/66/64/65/64/65	8/5/16/50/13/6/2/2/2/5			+
*trnP*	65/65/65/67/65/66/65/65/66				-
*nad6*	522/537/528/531/534/534/531/528/540	2/2/2/2/2/2/2/2/2/2	ATT/ATT/ATA/ATT/ATT/ATC/ATT/ATT/ATA	TAA/TAA/TAA/TAA/TAA/TAA/TAA/TAA/TAA	+
*cytb*	1152/1146/1149/1152/1152/1149/1152/1152/1152	8/-1/7/-1/6/30/-1/-1/-1/7	ATG/ATG/ATG/ATG/ATG/ATG/ATG/ATG/ATG	TAA/TAA/TAA/TAA/TAA/TAA/TAA/TAA/TAA	+
*trnS2*	63/65/68/69/65/66/69/66	-1/1/6/6/5/-2/4/2/-2/-2			+
*nad1*	939/939/939/936/936/942/939/942/942	-2/17/17/17/17/56/17/19/17/26	ATG/ATG/ATA/ATA/ATA/ATG/ATT/ATG/ATG	TAG/TAA/TAA/TAA/TAA/TAA/TAA/TAA/TAG	-
*trnL1*	67/68/68/68/68/69/68/67/69	9/1/1/-/3/3/1/-/-/1			-
*rrnL*	1359/1347/1359/1386/1379/1367/1390/1377/1363	49/18/14/-25/14/21/37/-21/-19/16			-
*trnV*	65/66/65/66/65/64/68/67/66	-/-/2/47/1/-/-2/2/-/2			-
*rrnS*	788/774/784/779/777/777/772/774/780	-1/-1/-1/-/-1/-/-1/-2/-/-1			-

## Data Availability

The following information was supplied regarding the availability of DNA sequences: The complete mitogenomes of *Abraximorpha davidii*, *Capila translucida*, *Celaenorrhinus aspersus*, *Erynnis popoviana*, *Gerosis phisara*, *Mooreana trichoneura*, *Pseudocoladenia dan fabia*, *Satarupa nymphalis*, *Tagiades menaka* are deposited in GenBank of NCBI under accession numbers MZ221157, MZ221158, MZ221159, MZ221165, MZ221160, MZ221161, MZ221162, MZ221163, and MZ221164, respectively.

## Supplementary Materials

The following supporting information can be downloaded at: https://www.mdpi.com/article/10.3390/insects13010068/s1, Figure S1: Predicted secondary cloverleaf structure for the tRNAs of *A. davidi*. Lines (-) indicate Watson-Crick base pairings, whereas dots (·) indicate unmatched base pairings. Figure S2: Predicted secondary cloverleaf structure for the tRNAs of *Ca. translucida*. Lines (-) indicate Watson-Crick base pairings, whereas dots (·) indicate unmatched base pairings. Figure S3: Predicted secondary cloverleaf structure for the tRNAs of *Ce. aspersus*. Lines (-) indicate Watson-Crick base pairings, whereas dots (·) indicate unmatched base pairings. Figure S4: Predicted secondary cloverleaf structure for the tRNAs of *E. popoviana*. Lines (-) indicate Watson-Crick base pairings, whereas dots (·) indicate unmatched base pairings. Figure S5: Predicted secondary cloverleaf structure for the tRNAs of *G. phisara*. Lines (-) indicate Watson-Crick base pairings, whereas dots (·) indicate unmatched base pairings. Figure S6: Predicted secondary cloverleaf structure for the tRNAs of *M. trichoneura*. Lines (-) indicate Watson-Crick base pairings, whereas dots (·) indicate unmatched base pairings. Figure S7: Predicted secondary cloverleaf structure for the tRNAs of *P. fabia*. Lines (-) indicate Watson-Crick base pairings, whereas dots (·) indicate unmatched base pairings. Figure S8: Predicted secondary cloverleaf structure for the tRNAs of *Sat. nymphalis*. Lines (-) indicate Watson-Crick base pairings, whereas dots (·) indicate unmatched base pairings. Figure S9: Predicted secondary cloverleaf structure for the tRNAs of *T. menaka*. Lines (-) indicate Watson-Crick base pairings, whereas dots (·) indicate unmatched base pairings. Figure S10: Phylogenetic tree produced by Bayesian inference analysis of the PCG dataset. Bayesian posterior probability (BPP) support values are indicated above the branches. Figure S11: Phylogenetic tree produced by maximum likelihood analyses of PCG dataset. Bootstrap support values (BS) are indicated above the branches. Figure S12: Phylogenetic tree produced by maximum likelihood analysis of the PRT dataset. Bootstrap support values (BS) are indicated above the branches. Figure S13: Phylogenetic tree produced by Bayesian inference analysis of the PCG12RT dataset. Bayesian posterior probability (BPP) support values are indicated above the branches. Figure S14: Phylogenetic tree produced by maximum likelihood analyses of PCG12RT dataset. Bootstrap support values (BS) are indicated above the branches. Figure S15: Phylogenetic tree produced by Bayesian inference analysis of the PRT dataset. Bayesian posterior probability (BPP) support values are indicated above the branches. Table S1: The basic statistics of sequencing for nine mitochondrial genomes. Table S2: The best partitioning schemes and models for Bayesian inference (BI) method based on the three datasets selected by PartitionFinder. Table S3: The best partitioning schemes and models for Maximun likelihood (ML) method based on three datasets selected by PartitionFinder.
