# Supplementary material for: Mitogenomes of Nine Asian Skipper Genera and Their Phylogenetic Position (Lepidoptera: Hesperiidae: Pyrginae)
Source: Insects. 2022 Jan 6;13(1):68. doi: 10.3390/insects13010068 (PMC8779469; doi:10.3390/insects13010068)
Supplement: Supplementary file 1 [file insects-13-00068-s001.zip › insects-1487564-supplementary.pdf]

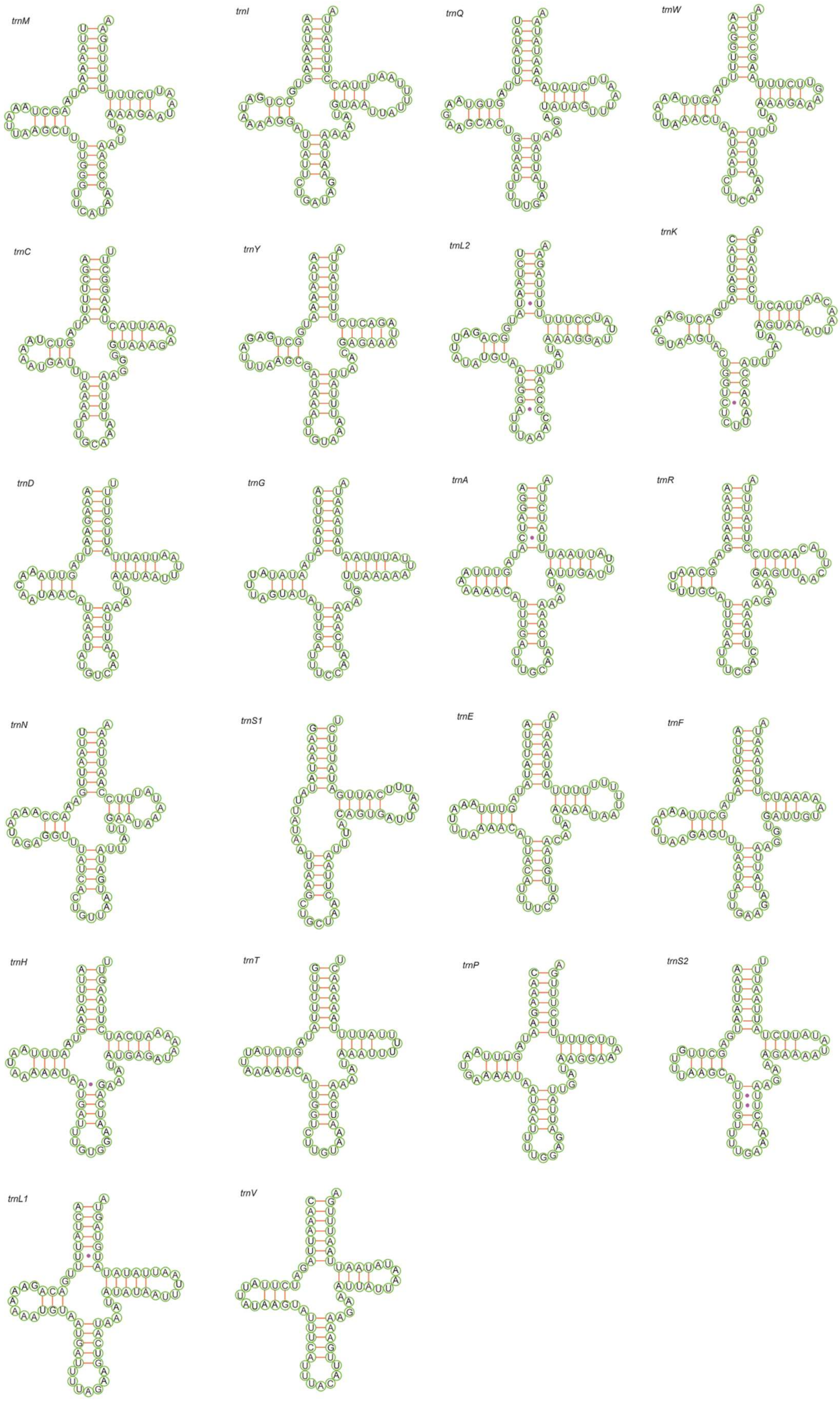

Figure S1. Predicted secondary cloverleaf structure for the tRNAs of *A. davidi*. Lines (-) indicate Watson-Crick base pairings, whereas dots (·) indicate unmatched base pairings.

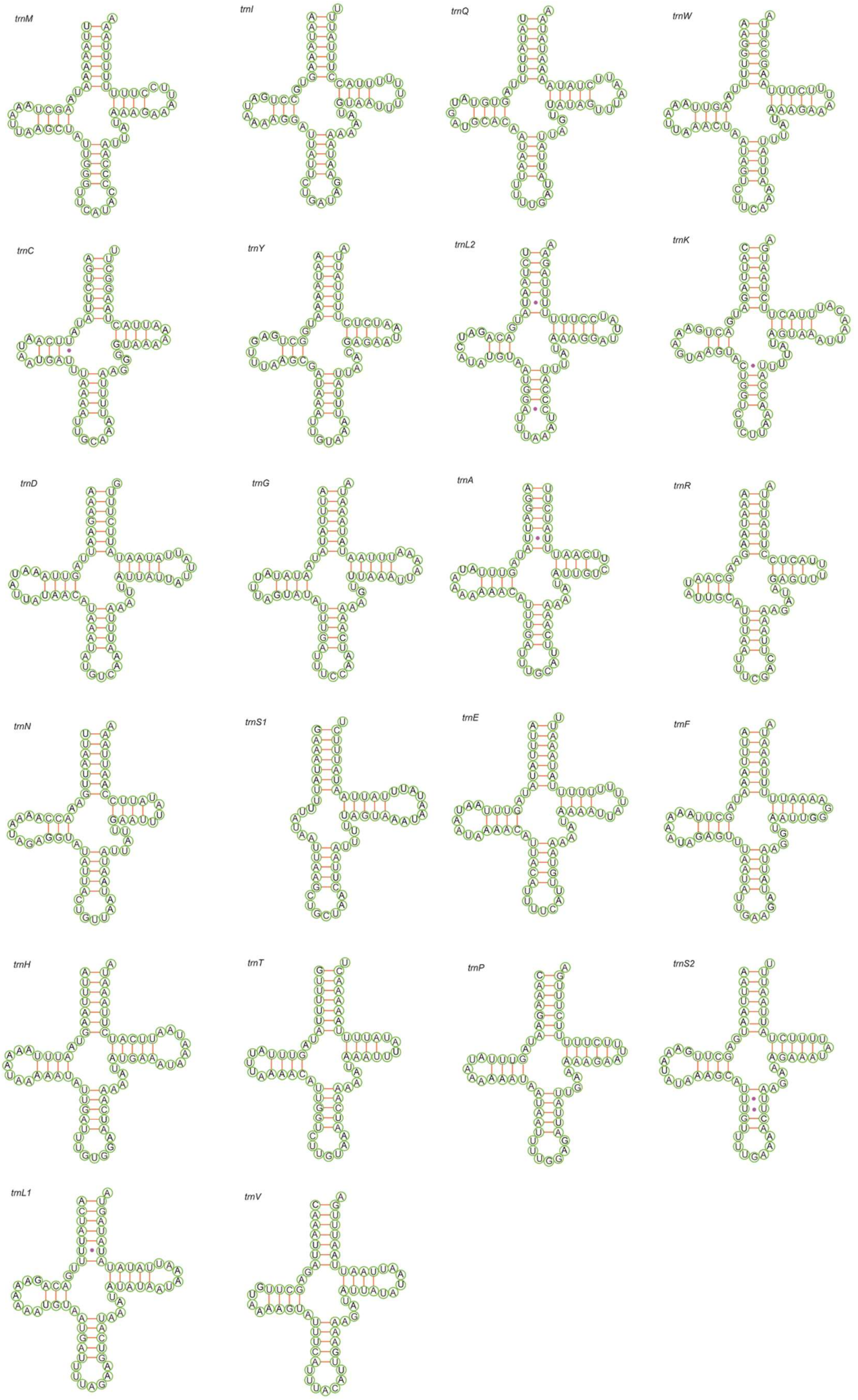

Figure S2. Predicted secondary cloverleaf structure for the tRNAs of *Ca. translucida*. Lines (-) indicate Watson-Crick base pairings, whereas dots (·) indicate unmatched base pairings.

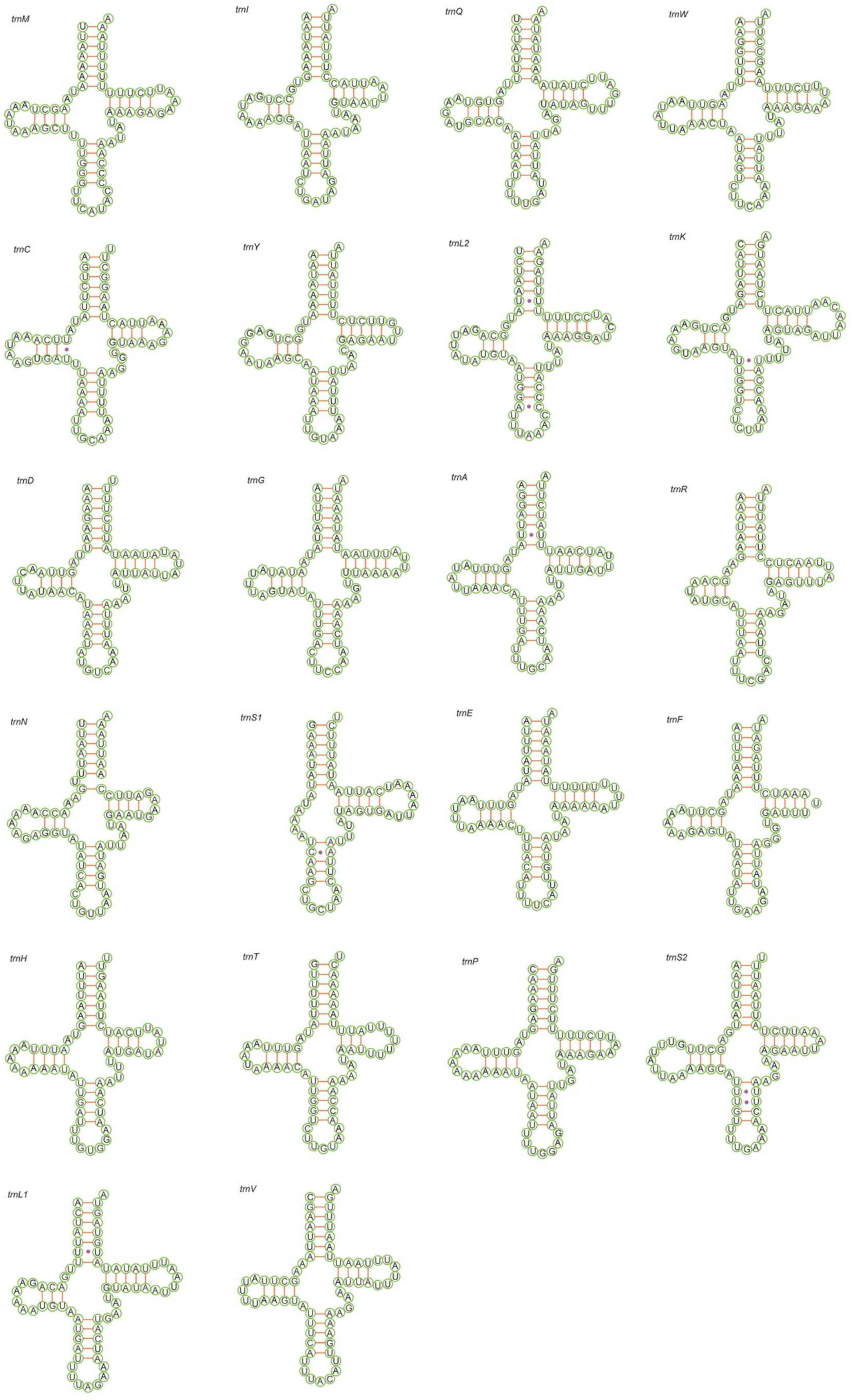

Figure S3. Predicted secondary cloverleaf structure for the tRNAs of *Ce. aspersus*. Lines (-) indicate Watson-Crick base pairings, whereas dots (·) indicate unmatched base pairings.

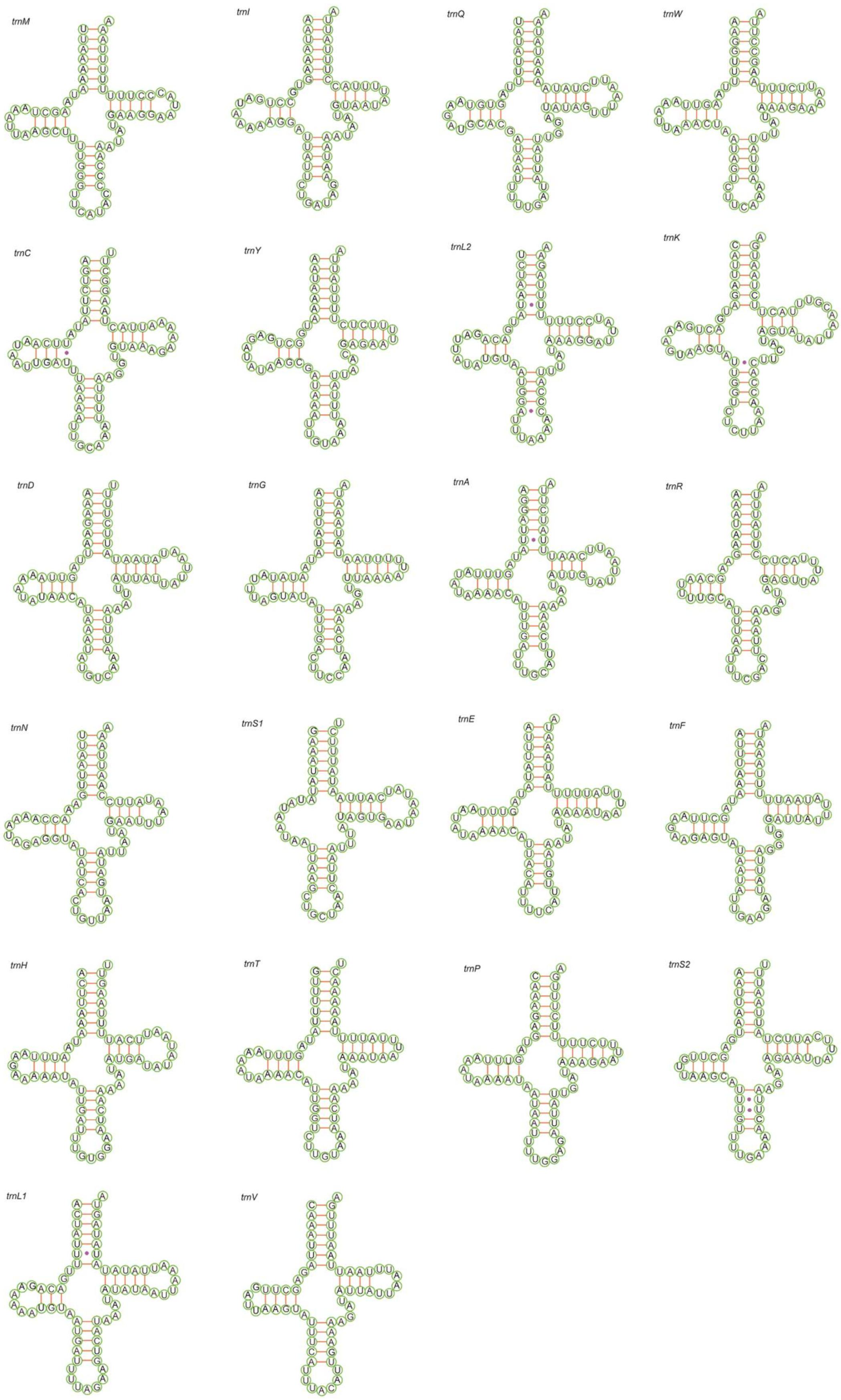

Figure S4. Predicted secondary cloverleaf structure for the tRNAs of *E. popoviana*. Lines (-) indicate Watson-Crick base pairings, whereas dots (·) indicate unmatched base pairings.

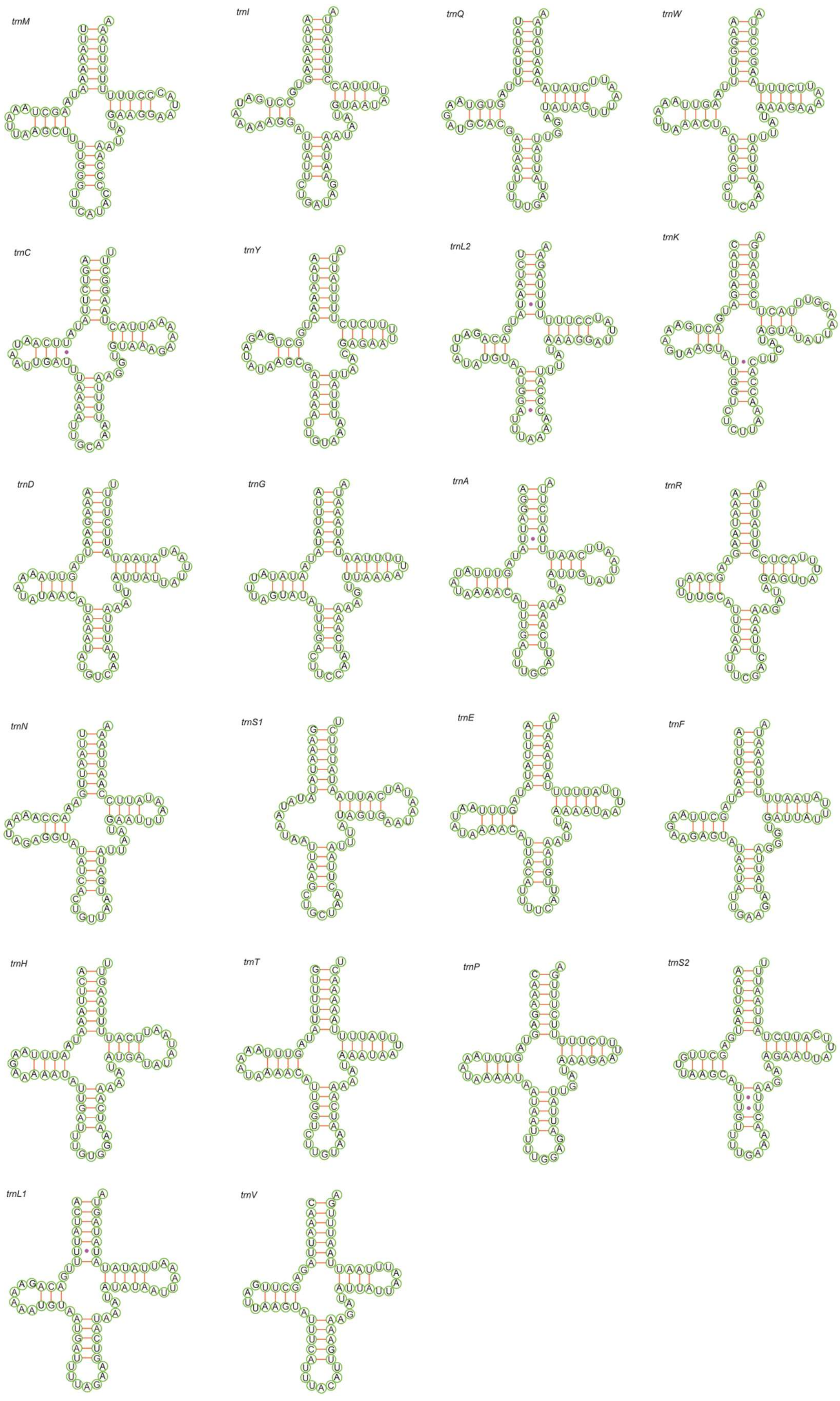

Figure S5. Predicted secondary cloverleaf structure for the tRNAs of *G. phisara*. Lines (-) indicate Watson-Crick base pairings, whereas dots (·) indicate unmatched base pairings.

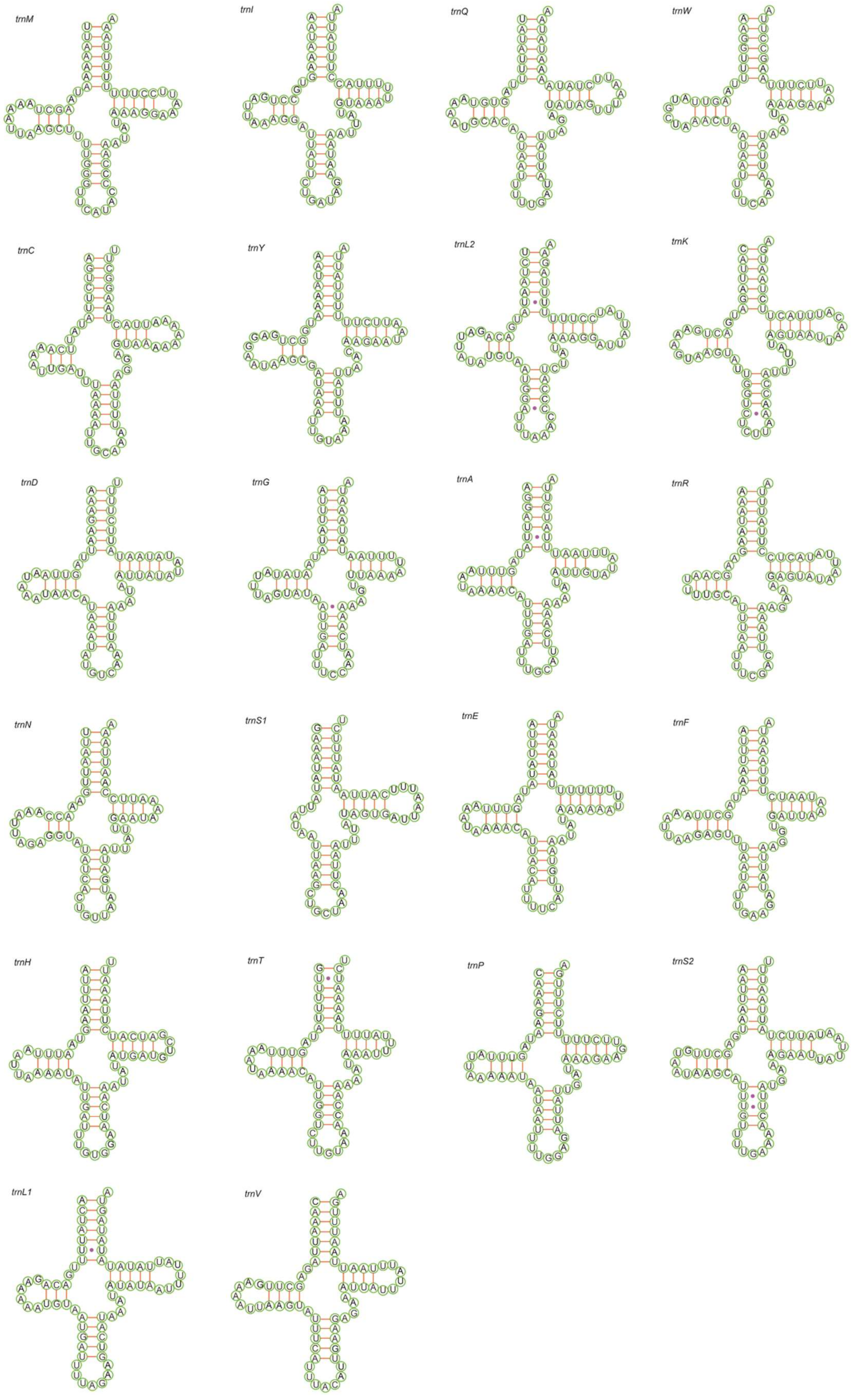

Figure S6. Predicted secondary cloverleaf structure for the tRNAs of *M. trichoneura*. Lines (-) indicate Watson-Crick base pairings, whereas dots (·) indicate unmatched base pairings.

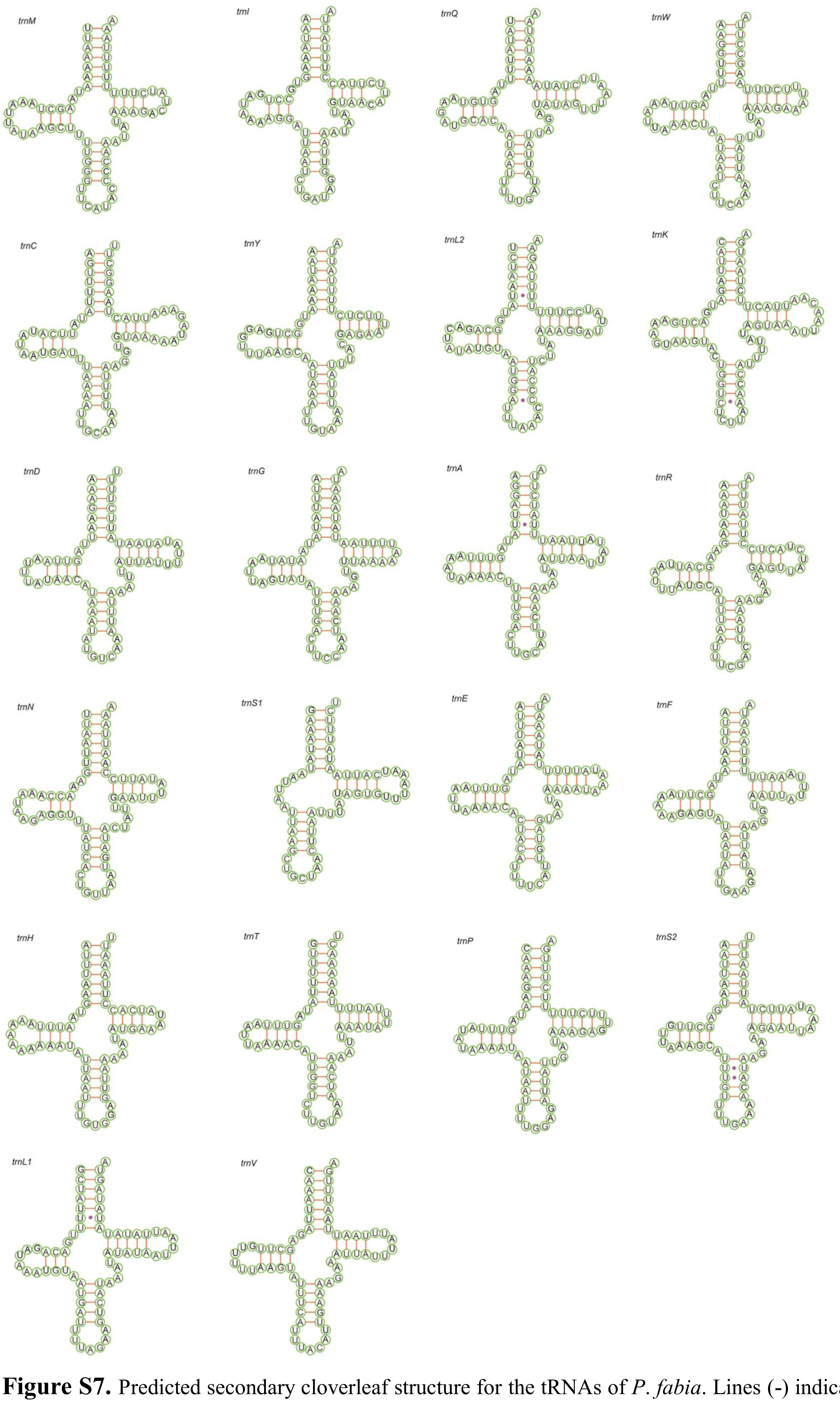

Figure S7. Predicted secondary cloverleaf structure for the tRNAs of *P. fabia*. Lines (-) indicate Watson-Crick base pairings, whereas dots (·) indicate unmatched base pairings.

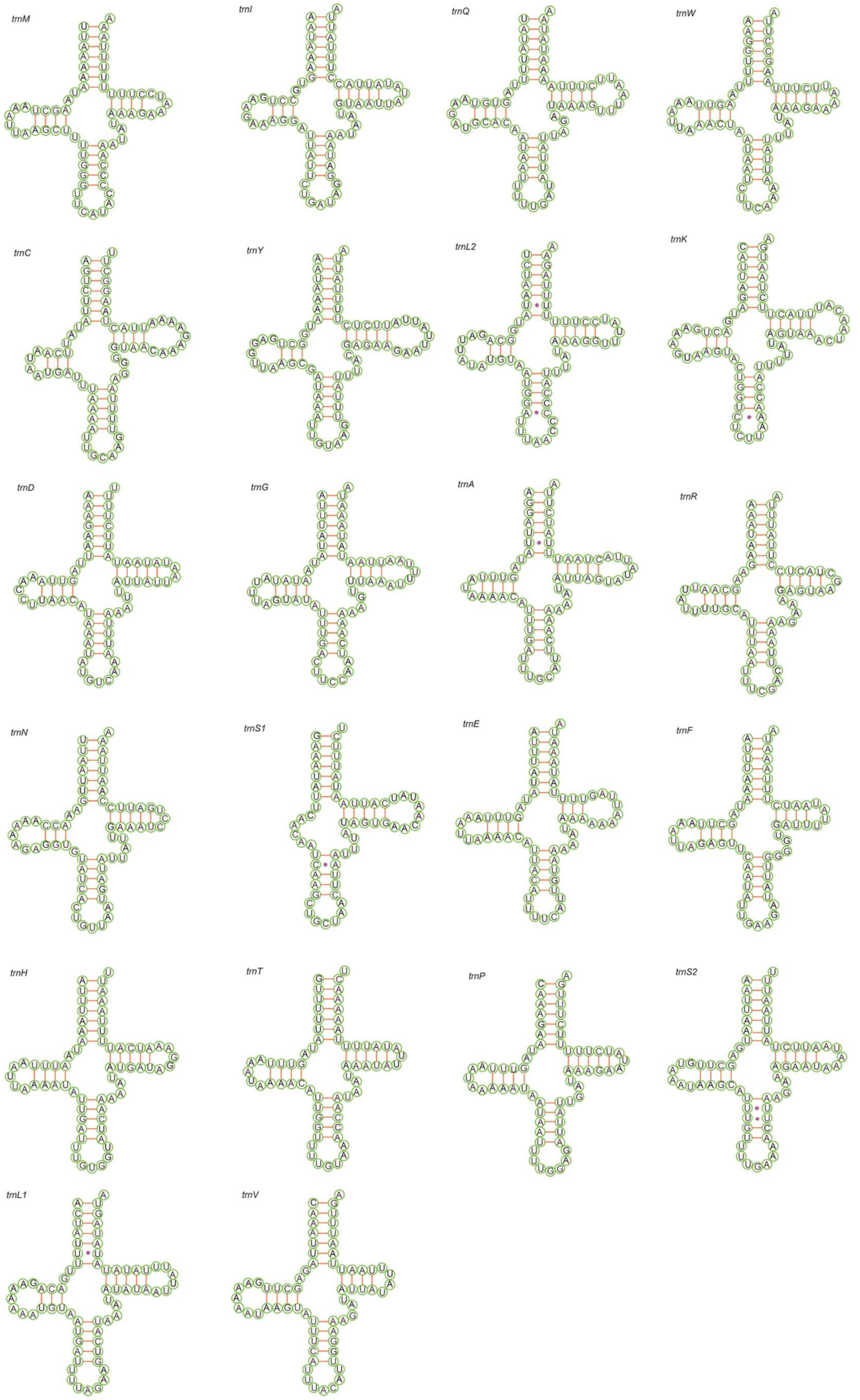

Figure S8. Predicted secondary cloverleaf structure for the tRNAs of *Sat. nymphalis*. Lines (-) indicate Watson-Crick base pairings, whereas dots (·) indicate unmatched base pairings.

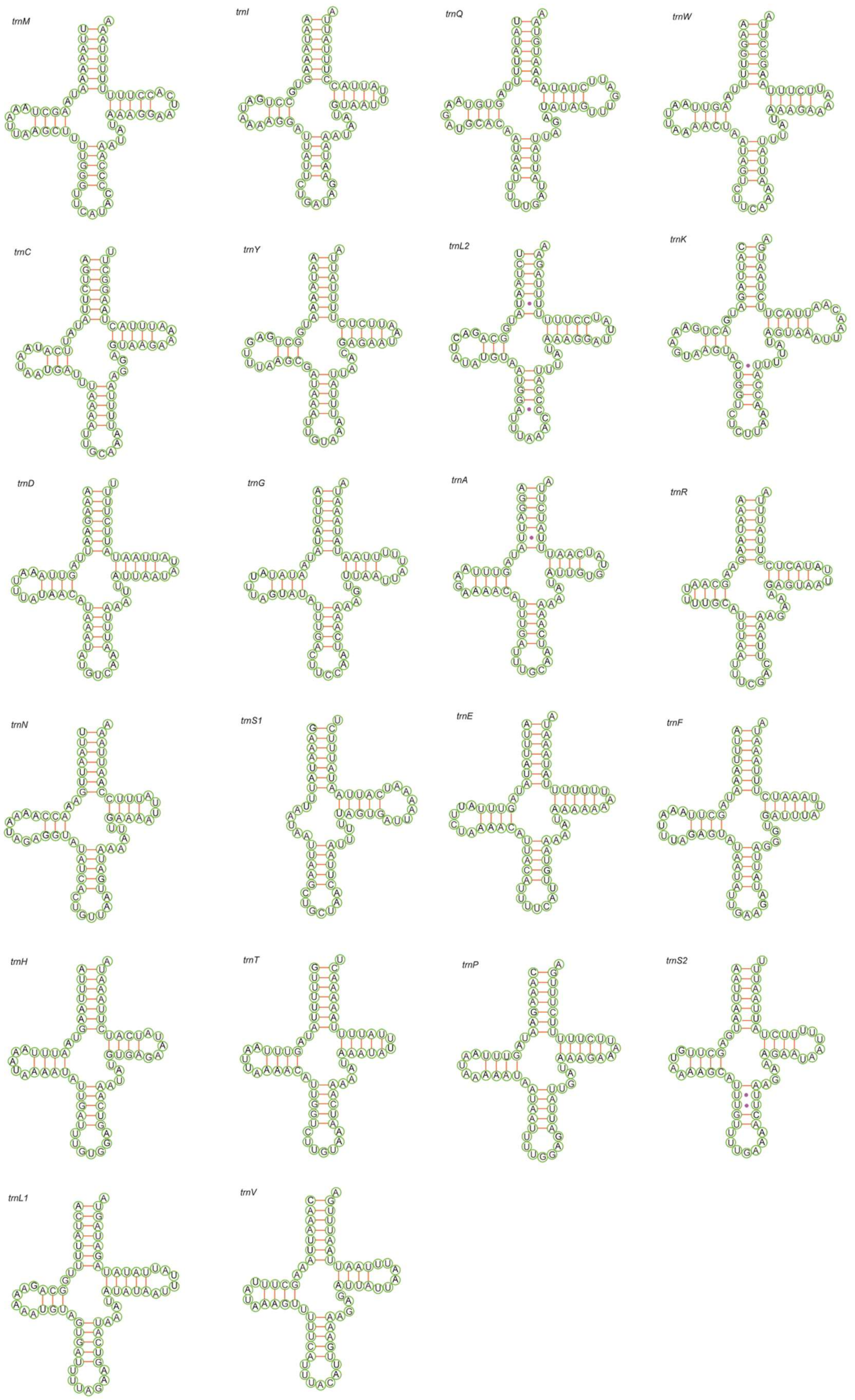

Figure S9. Predicted secondary cloverleaf structure for the tRNAs of *T. menaka*. Lines (-) indicate Watson-Crick base pairings, whereas dots (·) indicate unmatched base pairings.

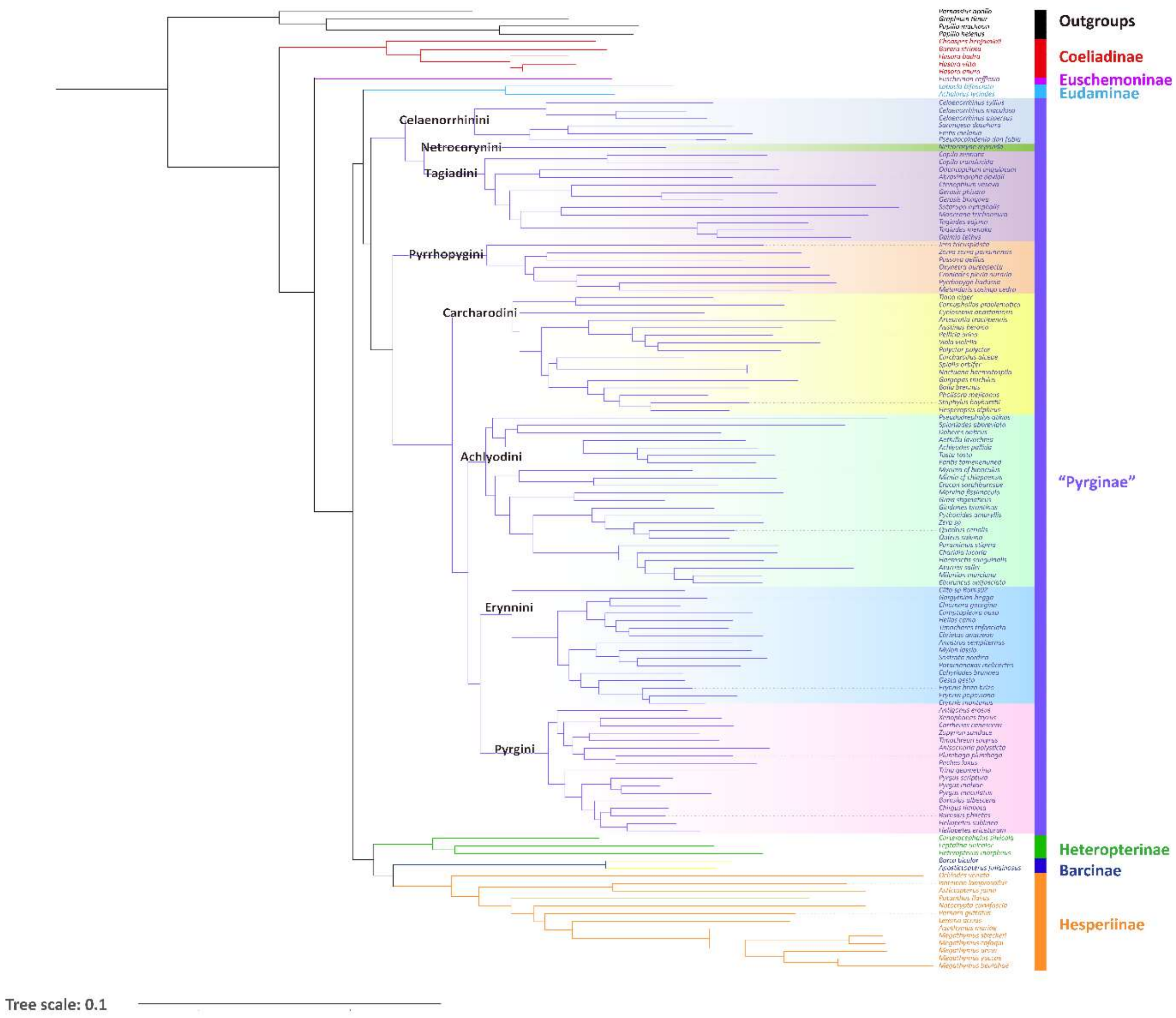

Figure S10. Phylogenetic tree produced by Bayesian inference analysis of the PCG dataset. Bayesian posterior probability (BPP) support values are indicated above the branches.

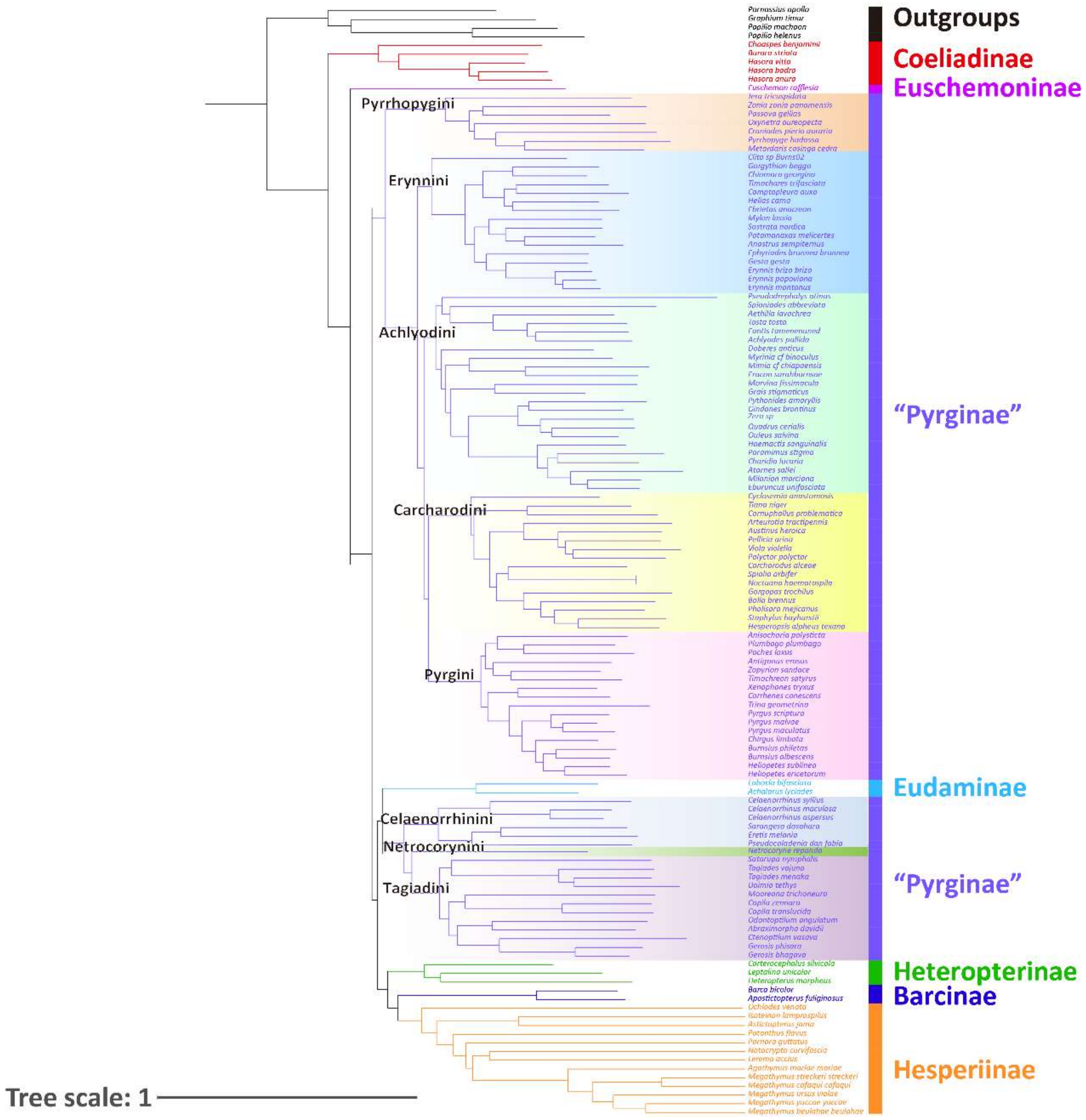

Figure S11. Phylogenetic tree produced by maximum likelihood analyses of PCG dataset. Bootstrap support values (BS) are indicated above the branches.

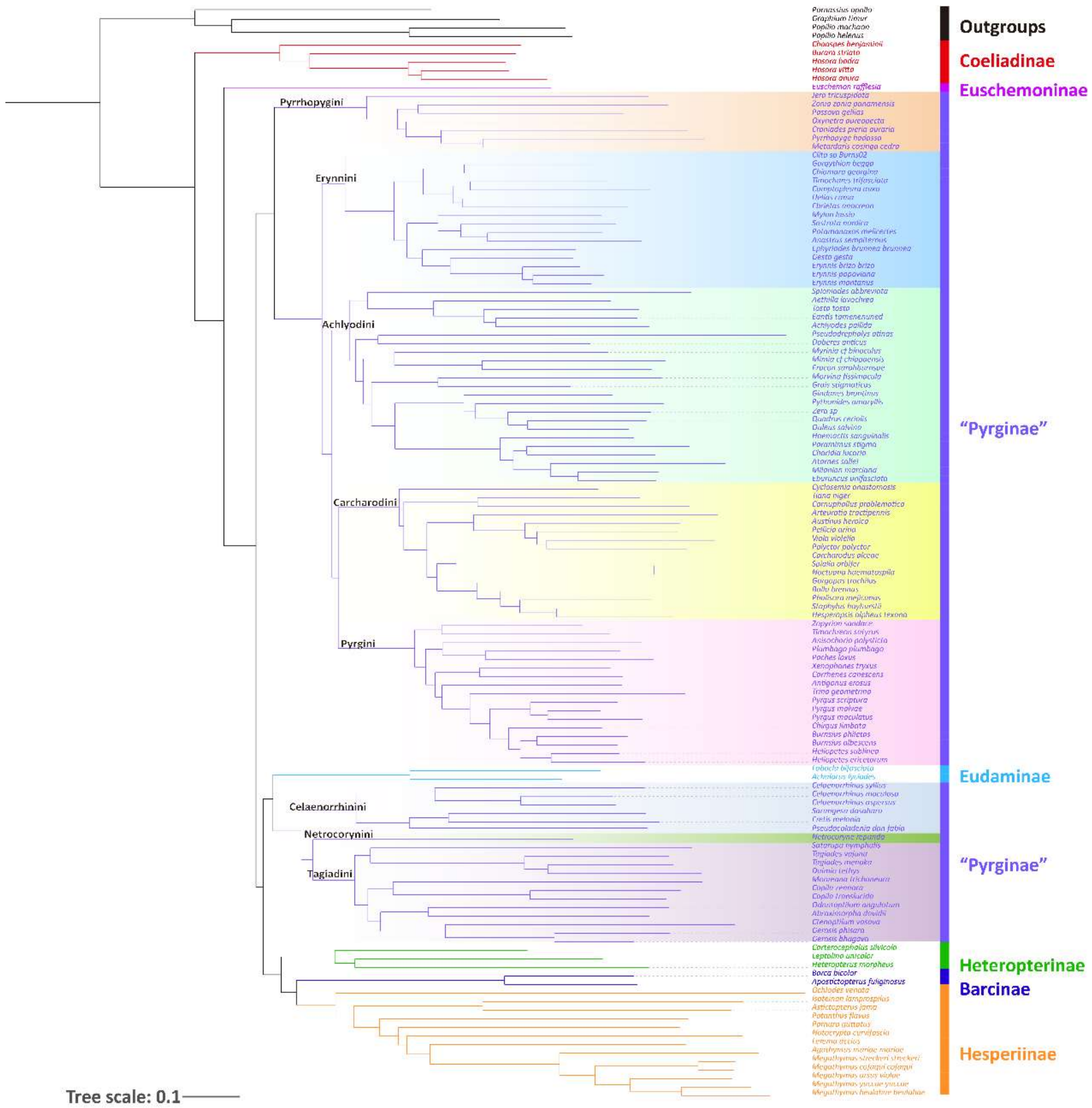

Figure S12. Phylogenetic tree produced by maximum likelihood analysis of the PRT dataset. Bootstrap support values (BS) are indicated above the branches.

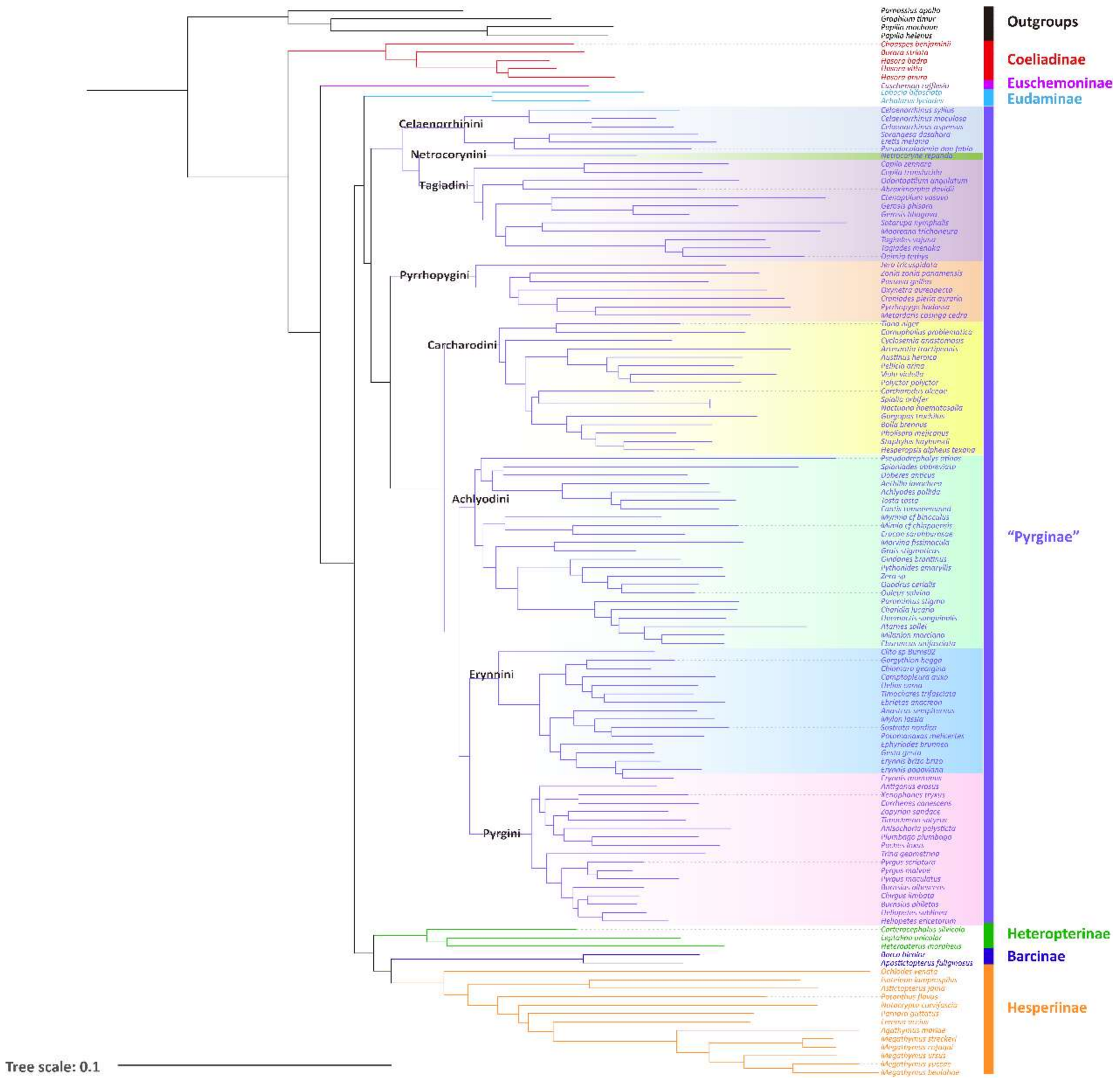

Figure S13. Phylogenetic tree produced by Bayesian inference analysis of the PCG12RT dataset. Bayesian posterior probability (BPP) support values are indicated above the branches.

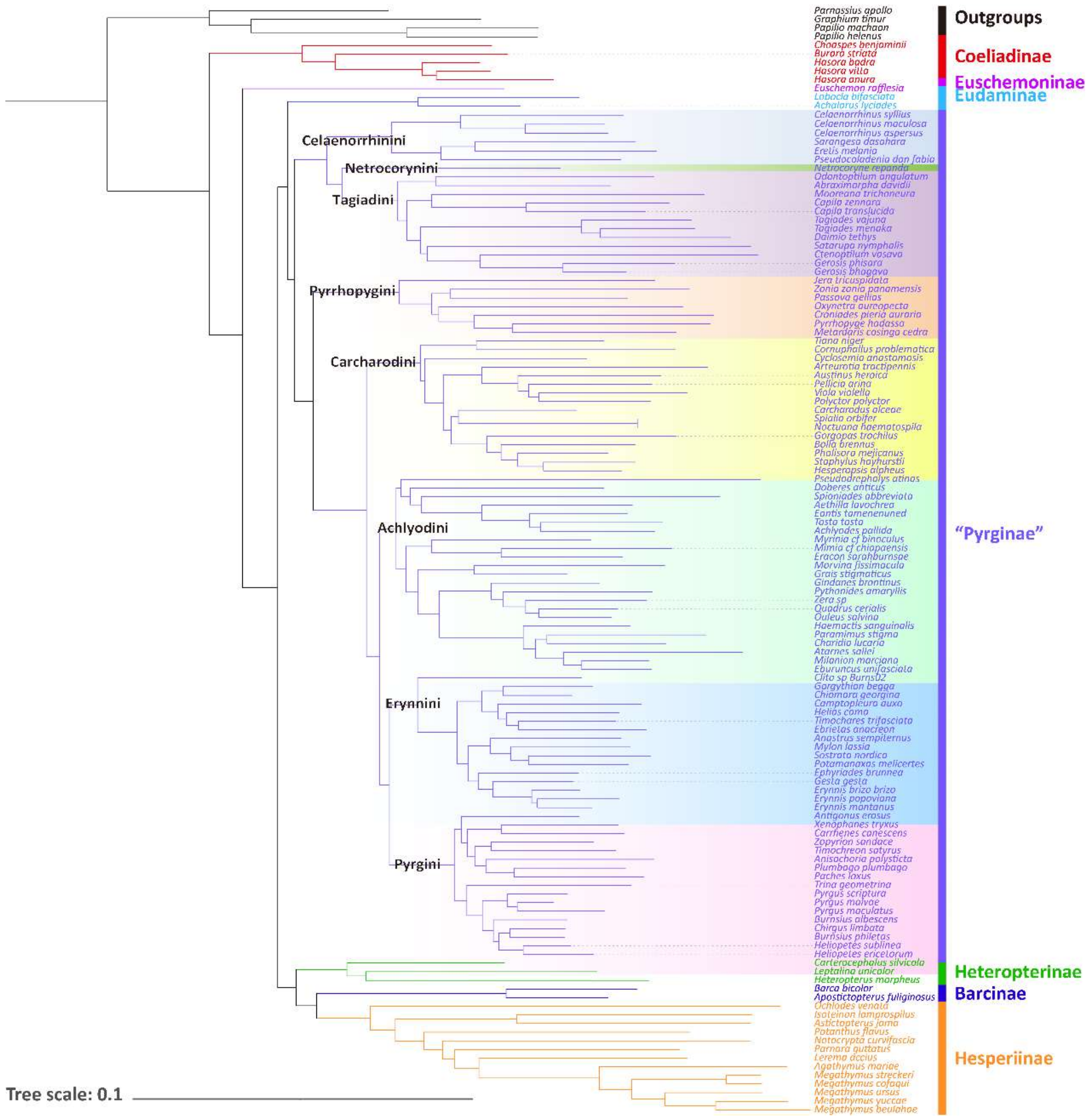

Figure S14. Phylogenetic tree produced by maximum likelihood analyses of PCG12RT dataset. Bootstrap support values (BS) are indicated above the branches.

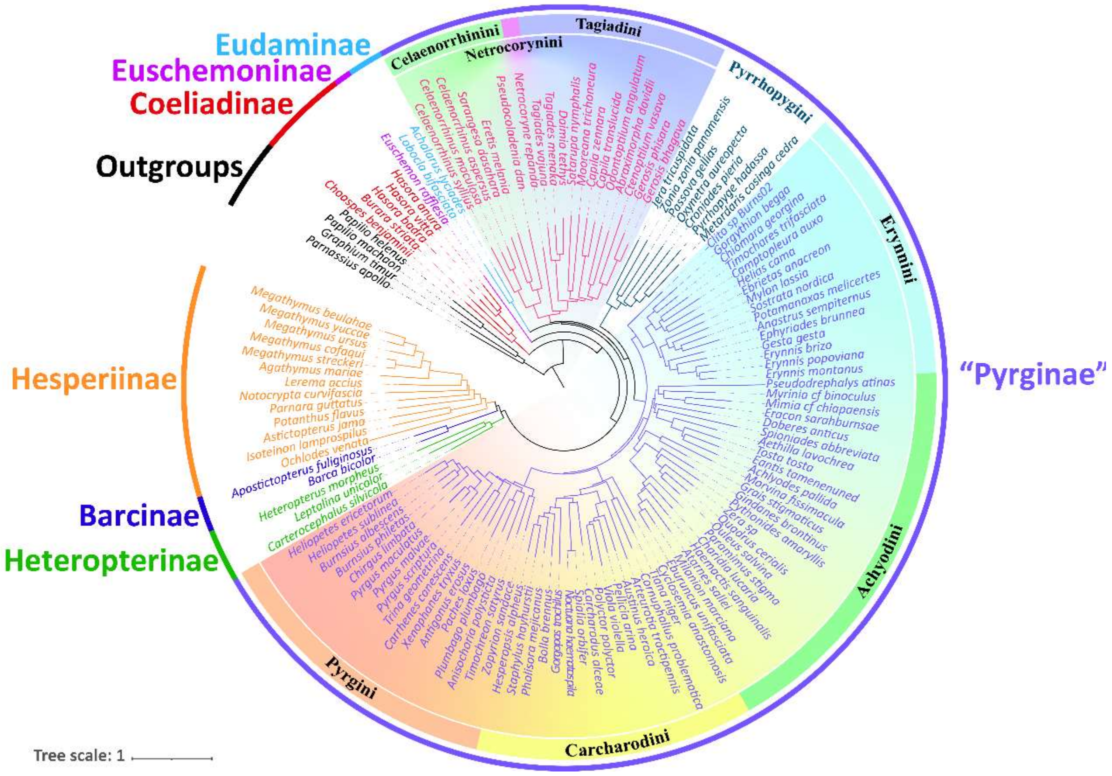

Figure S15. Phylogenetic tree produced by Bayesian inference analysis of the PRT dataset. Bayesian posterior probability (BPP) support values are indicated above the branches.

Table S1. The basic statistics of sequencing for nine mitochondrial genomes

Species	Sequence format	Raw reads	Clean reads
Abraximorpha davidii	Illumina	9,825,897	9,806,132
Capila translucida	Illumina	8,120,254	8,100,124
Celaenorrhinus aspersus	Illumina	9,147,121	9,137,060
Erynnis popoviana	Illumina	8,492,580	8,451,181
Gerosis phisara	Illumina	10,071,124	10,008,472
Mooreana trichoneura	Illumina	10,791,441	10,725,361
Pseudocoladenia dan fabia	Illumina	8,676,155	8,648,817
Satarupa nymphalis	Illumina	8,383,079	8,375,520
Tagiades menaka	Illumina	8,993,923	8,986,564

Table S2. The best partitioning schemes and models for Bayesian inference (BI) method based on three datasets selected by PartitionFinder.

Dataset	Partition	Subset Partitions	Model
**PCGs**	P1	*cox3*_pos3, *cytb*_pos3, *atp6*_pos3	GTR+I+G
	P2	*atp6*_pos2	GTR+I+G
	P3	*cytb*_pos3, *atp6*_pos3	HKY+G
	P4	*atp8*_pos3, *nad2*_pos3	GTR+I+G
	P5	*atp8*_pos2	GTR+I+G
	P6	*cox2*_pos3, *atp8*_pos3, *nad6*_pos3,	GTR+I+G
	P7	*cox1*_pos3	GTR+I+G
	P8	*cytb*_pos2, *cox1*_pos2, *cox2*_pos2	GTR+I+G
	P9	*cox1*_pos3	GTR+I+G
	P10	*cox2*_pos3	GTR+I+G
	P11	*nad6*_pos2, *cox3*_pos2, *nad3*_pos2, *nad2*_pos2	GTR+I+G
	P12	*nad3*_pos3, *cox3*_pos3	GTR+G
	P13	*nad4*_pos3, *nad4L*_pos3, *nad4L*_pos2, *nad1*_pos3. *nad5*_pos3	GTR+I+G
	P14	*nad4*_pos2, *nad1*_pos2, *nad5*_pos2	GTR+I+G
	P15	*nad1*_pos3	GTR+G
	P16	*nad3*_pos3, *nad2*_pos3	GTR+I+G
	P17	*nad4*_pos3, *nad4L*_pos3	GTR+G
	P18	*nad5*_pos3	HYK+I+G
	P19	*nad6*_pos3	GTR+I+G
**PRT**	P1	*cox3*_pos3, *cytb*_pos3, *atp6*_pos3	GTR+I+G
	P2	*atp6*_pos2	GTR+I+G
	P3	*cytb*_pos3, *atp6*_pos3, *cox1*_pos3	GTR+G
	P4	*atp8*_pos2, *atp8*_pos3, *trnS2*, *trnS1*	GTR+I+G

	P5	*atp8*_pos3, *cox2*_pos3	GTR+I+G
	P6	*cox2*_pos3, *cox1*_pos3	GTR+I+G
	P7	*cytb*_pos2, *cox1*_pos2, *cox2*_pos2	GTR+I+G
	P8	*nad6*_pos2, *cox3*_pos2, *nad3*_pos2, *nad2*_pos2	GTR+I+G
	P9	*nad6*_pos3, *nad3*_pos3, *cox3*_pos3	GTR+G
	P10	*nad1*_pos3, *nad5*_pos3	GTR+I+G
	P11	*nad4*_pos2, *nad1*_pos2, *nad5*_pos2	GTR+I+G
	P12	*nad1*_pos3	GTR+I+G
	P13	*nad3*_pos3, *trnA*, *trnT*, *trnE*, *nad2*_pos3	HKY+I+G
	P14	*nad2*_pos3	GTR+G
	P15	*trnN*, *trnM*, *trnL2*, *trnR*, *trnK*, *nad4*_pos3, *nad4L*_pos3	GTR+I+G
	P16	*trnH*, *trnG*, *trnY*, *nad4L*_pos2, *trnI*, *rrnS*, *trnD*, *trnV*, *rrnL*	GTR+I+G
	P17	*nad4L*_pos3, *nad5*_pos3	HKY+I+G
	P18	*nad4*_pos3	GTR+G
	P19	*nad6*_pos3	GTR+I+G
	P20	*trnQ*, *trnF*, *trnL1*, *trnC*, *trnP*, *trnW*	GTR+I+G
**12PRT**	P1	*atp6*, *cox3*, *cytb*	GTR+I+G
	P2	*atp8*	GTR+I+G
	P3	*cox1*	GTR+I+G
	P4	*cox2*	GTR+I+G
	P5	*nad1*, *nad5*	GTR+I+G
	P6	*nad3*, *nad2*, *trnE*, *trnT*, *trnA*	GTR+I+G
	P7	*trnG*, *trnW*, *trnY*, *trnP*, *trnV*, *rrnL*, *trnI*, *nad4L*, *rrnS*, *trnH*, *trnD*	GTR+I+G
	P8	*nad4*	GTR+I+G
	P9	*nad6*	GTR+I+G
	P10	*trnL1*, *trnQ*, *trnF*, *trnC*	HYK+G
	P11	*trnS2*, *trnS1*, *trnK*, *trnM*, *trnN*, *trnR*, *trnL2*	GTR+I+G

Table S3. The best partitioning schemes and models for Maximum likelihood (ML) method based on three datasets selected by PartitionFinder.

Dataset	Partition	Subset Partitions	Model
**PCGs**	P1	*cox3*_pos1, *cytb*_pos1, *atp6*_pos1	GTR+I+G
	P2	*atp6*_pos2	GTR+I+G
	P3	*cytb*_pos3, *atp6*_pos3	HKY+G
	P4	*atp8*_pos1, *atp8*_pos2, *nad3*_pos1, *nad6*_pos1	GTR+I+G
	P5	*atp8*_pos3, *nad6*_pos3, *cox2*_pos3	TRN+G
	P6	*cox1*_pos1	GTR+I+G
	P7	*cytb*_pos2, *cox1*_pos2, *cox2*_pos2	TVM+I+G
	P8	*cox1*_pos3	GTR+I+G
	P9	*cox2*_pos1	TIM+I+G
	P10	*nad6*_pos2, *cox3*_pos2, *nad3*_pos2, *nad2*_pos2	GTR+I+G
	P11	*nad3*_pos3, *cox3*_pos3	TVM+G
	P12	*nad4*_pos1, *nad4L*_pos1, *nad4L*_pos2, *nad1*_pos1, *nad5*_pos1	GTR+I+G
	P13	*nad4*_pos2, *nad1*_pos2, *nad5*_pos2	GTR+I+G
	P14	*nad1*_pos3	TIM+G
	P15	*nad2*_pos1	TRN+I+G
	P16	*nad2*_pos3	K81UF+G
	P17	*nad4*_pos3, *nad4L*_pos3	TIM+G
	P18	*nad5*_pos3	HKY+I+G
**12PRT**	P1	*atp6_mafft_gb, cox3_mafft_gb, cytb_mafft_gb*	GTR+I+G
	P2	*atp8_mafft_gb*	GTR+I+G
	P3	*cox1_mafft_gb*	GTR+I+G
	P4	*cox2_mafft_gb*	TIM+I+G
	P5	*nad1_mafft_gb, nad5_mafft_gb*	GTR+I+G
	P6	*nad3_mafft_gb, nad2_mafft_gb, trnE_gb, trnT_gb, trnA_gb*	TIM+I+G
	P7	*trnG_gb, trnW_gb, trnY_gb, trnP_gb, trnV_gb, rrnL_gb, trnI_gb, nad4L_mafft_gb, rrnS_gb, trnH_gb, trnD_gb*	GTR+I+G
	P8	*nad4_mafft_gb*	TIM+I+G
	P9	*nad6_mafft_gb*	GTR+I+G
	P10	*trnL1_gb, trnQ_gb, trnF_gb, trnC_gb*	TRN+G
	P11	*trnS2_gb, trnS1_gb, trnK_gb, trnM_gb, trnN_gb, trnR_gb, trnL2_gb*	TVM+I+G
**PRT**	P1	*cox3*_pos1, *cytb*_pos1, *atp6*_pos1	GTR+I+G
	P2	*atp6*_pos2	GTR+I+G
	P3	*atp6*_pos3	TRN+G
	P4	*nad6*_pos1, *atp8*_pos1	GTR+I+G
	P5	*trnS2_gb, atp8*_pos2, *trnE_gb, trnS1_gb*	GTR+I+G
	P6	*atp8*_pos3, *cytb*_pos3	TRN+I+G
	P7	*cox1*_pos1	GTR+I+G

P8	*cox1*_pos2, *cox2*_pos2	TVM+I+G
P9	*nad2*_pos3, *cox1*_pos3	K81UF+I+G
P10	*cox2*_pos1	TIM+I+G
P11	*cox2*_pos3, *nad6*_pos3	TIM+G
P12	*cytb*_pos2, *cox3*_pos2, *nad3*_pos2, *nad2*_pos2	GTR+I+G
P13	*nad3*_pos3, *cox3*_pos3	TIM+G
P14	*nad1*_pos1, *nad5*_pos1	GTR+I+G
P15	*nad4*_pos2, *nad1*_pos2, *nad5*_pos2	GTR+I+G
P16	*nad1*_pos3	TIM+G
P17	*nad2*_pos1	TRN+I+G
P18	*nad3*_pos1, *trnA_gb, trnT_gb*	GTR+I+G
P19	*trnN_gb, trnM_gb, trnL2_gb, trnR_gb, trnK_gb,* *nad4*_pos1, *nad4L_codon*	GTR+I+G
P20	*trnH_gb, trnD_gb, trnY_gb, nad4L*_pos2, *trnI_gb, rrnS_gb, rrnL_gb, trnV_gb*	GTR+I+G
P21	*nad4L*_pos3, *nad4*_pos3	TIM+G
P22	*nad5*_pos3	K81UF+I+G
P23	*nad6*_pos2	TVM+I+G
P24	*trnG_gb, trnL1_gb, trnP_gb, trnW_gb, trnQ_gb, trnF_gb, trnC_gb*	TIM+I+G
